# Comparative Analysis of the Complete Chloroplast Genome of Four Endangered Herbals of *Notopterygium*

**DOI:** 10.3390/genes8040124

**Published:** 2017-04-19

**Authors:** Jiao Yang, Ming Yue, Chuan Niu, Xiong-Feng Ma, Zhong-Hu Li

**Affiliations:** 1 Key Laboratory of Resource Biology and Biotechnology in Western China, Ministry of Education, College of Life Sciences, Northwest University, Xi’an 710069, China; yangjiao0818@163.com (J.Y.); yueming@nwu.edu.cn (M.Y.); 15829726401@163.com (C.N.); 2 State Key Laboratory of Cotton Biology, Institute of Cotton Research, Chinese Academy of Agricultural Sciences, Anyang 455000, China

**Keywords:** chloroplast genome, evolutionary rate, Illumina sequencing, *Notopterygium*, phylogenetic relationship

## Abstract

*Notopterygium* H. de Boissieu (Apiaceae) is an endangered perennial herb endemic to China. A good knowledge of phylogenetic evolution and population genomics is conducive to the establishment of effective management and conservation strategies of the genus *Notopterygium*. In this study, the complete chloroplast (cp) genomes of four *Notopterygium* species (*N. incisum* C. C. Ting ex H. T. Chang, *N. oviforme* R. H. Shan, *N. franchetii* H. de Boissieu and *N. forrestii* H. Wolff) were assembled and characterized using next-generation sequencing. We investigated the gene organization, order, size and repeat sequences of the cp genome and constructed the phylogenetic relationships of *Notopterygium* species based on the chloroplast DNA and nuclear internal transcribed spacer (ITS) sequences. Comparative analysis of plastid genome showed that the cp DNA are the standard double-stranded molecule, ranging from 157,462 bp (*N. oviforme*) to 159,607 bp (*N. forrestii*) in length. The circular DNA each contained a large single-copy (LSC) region, a small single-copy (SSC) region, and a pair of inverted repeats (IRs). The cp DNA of four species contained 85 protein-coding genes, 37 transfer RNA (tRNA) genes and 8 ribosomal RNA (rRNA) genes, respectively. We determined the marked conservation of gene content and sequence evolutionary rate in the cp genome of four *Notopterygium* species. Three genes (*psaI*, *psbI* and *rpoA*) were possibly under positive selection among the four sampled species. Phylogenetic analysis showed that four *Notopterygium* species formed a monophyletic clade with high bootstrap support. However, the inconsistent interspecific relationships with the genus *Notopterygium* were identified between the cp DNA and ITS markers. The incomplete lineage sorting, convergence evolution or hybridization, gene infiltration and different sampling strategies among species may have caused the incongruence between the nuclear and cp DNA relationships. The present results suggested that *Notopterygium* species may have experienced a complex evolutionary history and speciation process.

## 1. Introduction

*Notopterygium* H. de Boissieu (Apiaceae) is an endangered perennial herb endemic to China [1]. According to the records of ‘Flora of China’, six species are included in the genus *Notopterygium*, *N. incisum* C. C. Ting ex H. T. Chang, *N. oviforme* R. H. Shan, *N. franchetii* H. de Boissieu, *N. forrestii* H. Wolff, *N. tenuifolium* M. L. Sheh and F. T. Pu and *N. pinnatiinvolucellum* F. T. Pu and Y. P. Wang. As an important traditional Chinese medicinal plant, the species of *Notopterygium* are generally used for treating cold headache, rheumatism paralysis, and shoulder joint ache. Meanwhile, *N. incisum* and *N. franchetii* also have other pharmacological effects, including anti-inflammation, anti-bacterial, anti-allergic, anti-viral, etc. [2]. *Notopterygium* species are geographically distributed among mid-to high-elevation regions (1700 to 5000 m) and are mainly distributed in the alpine mountains of the Qinghai-Tibetan Plateau and adjacent regions [3]. The previous studies on the *Notopterygium* species mainly focused on their morphology, anatomy [4], systematics [5], ecology [6] and pharmacognosy [7]. In recent years, excavation, as well as the expansion of human activities and climate oscillations, has resulted in the increasingly declining wild populations of the *Notopterygium* species. Now, the natural populations of these *Notopterygium* species are severely fragmented, and need urgent conservation and restoration [8]. A good knowledge of phylogenetic evolution and population genomics is very important to formulate effective conservation and management strategies. In recent years, comparative analysis of the complete chloroplast (cp) genome of different close species has provided a promising method for the study of phylogeny, population dynamics and species evolution [9,10,11].

In general, the cp genome of angiosperms is a circular molecule of double-stranded DNA ranging from 76 to 217 kb in size and containing about 130 genes [12,13,14]. Gene structure, content, and order of the cp DNA are often highly conserved, and generally contain two identical copies of inverted repeats (IRs), separating the large single-copy (LSC) region and the small single-copy (SSC) region [15,16,17]. The complete cp DNA is useful in plant systematics and evolution research because of its maternal inheritance and conserved structure characteristics [17]. Recently, many sequences of the complete cp genome of higher plants have been reported [18], which were used to study plant molecular identification, population structure, and phylogenetic relationships at low to high taxonomic levels [19,20,21,22]. Especially, the comparative analysis of cp genomes among closely related species shows considerable promise for studying the evolutionary history and species conservation and increasing understanding of phylogenetic relationships [23,24,25,26].

The traditional method of Sanger sequencing was troublesome, time-consuming and difficult for isolating and sequencing complete cp genome [27]. In recent years, with the rapid development of high-throughput sequencing technology, more and more complete plant cp DNA sequences have been constructed based on next generation sequencing (NGS) methods [21,22]. Meanwhile, Illumina-based plastid sequencing has been proven to be a valid and cost-effective way to acquire the complete cp DNA. Many assembled cp DNA of non-model species also have been obtained and employed for the study of differential gene expression, genetic markers development [28], phylogenomics analysis [29], as well as for detecting selection and inferring adaptive evolution within and among angiosperm species [30,31].

In this study, we used an Illumina Miseq sequencing platform to obtain the whole cp DNA sequence of four endangered herbal plants in the genus *Notopterygium*, *N. incisum*, *N. oviforme*, *N. franchetii* and *N. forrestii*. Then, in order to obtain a comprehensive and deeply understanding of evolutionary relationships and speciation progress, we also sequenced the nuclear internal transcribed spacer (ITS) sequences and constructed the interspecific relationships among *Notopterygium* species. The comparative analysis of cp DNA and nuclear ITS sequence variations will provide the basic knowledge for study of the complex evolutionary process and phylogeny for these endangered herbal species.

## 2. Materials and Methods

### 2.1. Plant Material and DNA Extraction

For chloroplast genome analyses, fresh leaves of *N. incisum*, *N. oviforme*, *N. franchetii* and *N. forrestii* were collected in the field in July, 2015. Total genomic DNA of four individuals from four *Notopterygium* species were isolated using modified 4xCTAB method [32]. For ITS analyses, the dried leaves of 70 individuals of eight populations from four species were also collected (Supplementary, Table S1).

### 2.2. Genome Sequencing, Assembly and Annotation

After the genomic DNA isolation, approximately 5–10 μg of DNA was sheared, followed by adapter ligation and library amplification. Then, the fragmented DNAs were subjected to Illumina sample preparation, and pair-read sequenced conducted on the Illumina Miseq platform by Novo Gene Bioinformatics Technology Co. Ltd. (Beijing, China). Illumina raw reads (*N. incisum* SRR5438310, *N. oviforme* SRR5438311, *N. franchetii* SRR5436396, and *N. forrestii* SRR5436275) were firstly quality trimmed using the Trimming function in NGSQCToolkit v2.3.3 [33]. After low quality reads and adapters were dislodged, the clean reads were firstly assembled using MIRA v4.0.2 [34] with the cp genome of closely related species *Anthriscus cerefolium* (GU456628), *Daucus carota* (DQ898156) and *Petrosilenum crispum* (HM596073) as the references. Subsequently, the desirable contigs were further assembled using MITObim-master [35]. To obtain more accurate cp DNA sequences, each species was assembled at least three times using different references, respectively. In addition, we also used the CLC Genomics workbench v6.5 software with the default parameters set (CLG Bio, Aarhus, Denmark) to assemble the raw reads of four *Notopterygium* species. We conducted a de novo assembly in order to verify the validity and accuracy of assembly results. After assembled, the obtained scaffolds and contigs were aligned and compared to the reference-based assembly. We also checked the assemblies with read mapping and found that they were not forcing the reads into a false structure. Then, the obtained contigs were generated a consensus sequence in the Geneious R8 v8.0.2 (Biomatters Ltd., Auckland, New Zealand). The gaps were filled by realignment of input reads. Meanwhile, some ambiguous regions with low coverage were confirmed by mapped with reference. We also checked the published complete cp genome sequences available for *A. cerefolium*, *P. crispum*, and *D. carota* using the program MAFFT [36].

In addition, in order to further validate the accuracy of assembly results of cp DNA, we carried out Sanger sequencing of some cp DNA gene regions, e.g., *matk*, *rbcL*, *trnS-trnG* (with accession numbers: KY924852-KY924863). We found that these newly obtained cp DNA sequences were highly matched with assembled chloroplast genome. Then, the complete cp genome was annotated using the program DOGMA and manually adjusted the start and stop codons in Geneious R8 [37]. Eventually, a circular cp DNA map was drawn with the online program Organellar Genome DRAW v1.1 (OGDRAW) [38]. The newly obtained cp genome of four species, *N. incisum*, *N. oviforme*, *N. franchetii* and *N. forrestii* were deposited in GenBank under accession number KX808491-KX808494.

### 2.3. Genome Repeat, Gene Selective Pressure Analysis, Phylogenetic Analysis

We determined the four types of repeats among four cp DNA sequences: dispersed, palindromic, tandem and transfer RNA (tRNA) or gene-similar, the first and second types applying the program REPuter [39], the third one applying the online program Tandem Repeats Finder [40], and the fourth type using the manual revision. The relative evolutionary rate of the cp DNA sequence was quantified based on nonsynonymous (dN) and synonymous (dS) substitutions and their ratios (ω = dN/dS). dN and dS were computed according to the LWL85, LPB93 & LWLm methods [41,42,43] as implemented in the PAML package v4.7.1 [44] using the F3 × 4 codon-based substitution model. Only codons shared among all cp genomes were compared. dN, dS and ω were calculated for (1) individual protein-coding genes; (2) the combinations of all protein-coding genes; (3) groups of genes with the same functions, e.g., photosynthesis ATP-synthase genes or NADH-dehydrogenase genes [45]. There were six pairwise alignments for each gene region, which contributed to a total of 283 ω values.

In order to detect the interspecific relationships among four *Notopterygium* species, we also added the ten cp genomes of the Apiaceae family available in GenBank. Five Araliaceae cp genomes were also downloaded as outgroups (Table S2). We used the following six partitions datasets to conduct a maximum likelihood (ML) tree by using the program MEGA v7.0.18 [46]: (A) whole cp genomes; (B) all data excluding a IR region; (C) the LSC region; (D) the SSC region; (E) the protein-coding sequences; (F) the IR region. In addition, the nuclear ITS sequences of 8 populations from four *Notopterygium* species were sequenced. The obtained 15 ITS (KY848833-KY848847) haplotypes were used to construct phylogenetic tree based on Bayesian inference (BI) [47] and ML analyses [48], using *Heracleum moellendorffii*, *Pleurospermum franchetianum* and *Pleurospermum prattii* as outgroup (KY848848-KY848850). Based on the Akaike information criterion (AIC) in jModeltest v2.1.3 [49], the best-fitting models of sequence evolution were GTR+G (ITS) and GTR+G+I (cp genome), respectively.

### 2.4. Sequence Divergence Analysis

The cp DNA rearrangement analyses of four *Notopterygium* species were performed using Mauve Alignment [50]. In order to show interspecific variation, the alignments of the cp DNA of the four *Notopterygium* species were visualized by mVISTA and *N. incisum* as a reference [51]. The percentages of variable characters for coding and noncoding regions were computed based on the method of Zhang et al. [52]. Nucleotide substitutions were counted using MEGA v7.0.18 [46], and indels (insertion/deletion) were manually detected across the cp genomes.

## 3. Results

### 3.1. The Overall Features of cp DNA of Four Notopterygium Species

**Figure 1:**
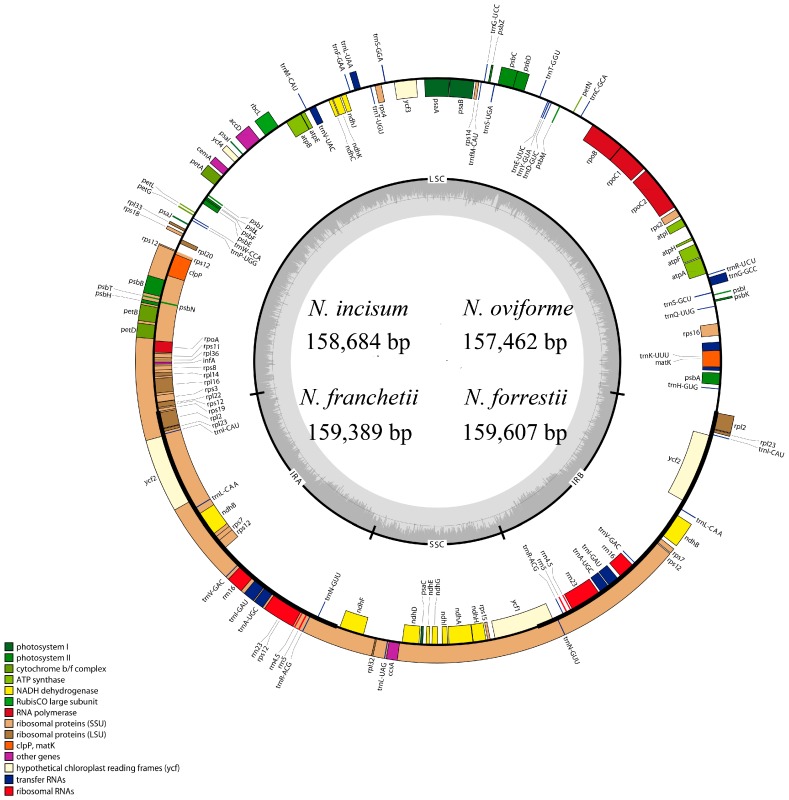
Gene map of four *Notopterygium* species. Genes lying outside of the outer layer circle are transcribed in the counter clockwise direction, whereas genes inside are transcribed in the clockwise direction. The colored bars indicate known different functional groups. The darker gray area in the inner circle denotes GC content while the lighter gray corresponds to AT content of the genome. LSC: large single copy, SSC: small-single-copy, IR: inverted repeat.

The assembled reads of four *Notopterygium* species, arranged from a total of 110,460 reads of *N. franchetii* to 375,226 reads of *N. incisum* (Table S3). The complete cp DNA of the four *Notopterygium* species was a double-stranded molecule from 157,462 bp of *N. oviforme* to 159,607 bp of *N. forrestii* in length. The cp genome had an overall typical quadripartite structure that was similar to the majority of land plant cp genomes, and contained a pair of IR regions (IRa and IRb), one LSC region and one SSC region. The GC contents of the LSC, SSC, IR regions, and the whole cp genome of four species is shown in Table 1 and Figure 1. The cp genomes of *N. incisum*, *N. franchetii* and *N. forrestii* had the same GC content (37.70%), *N. oviforme* had a subtle difference (37.90%) compared with others. The GC contents of the LSC and SSC regions (35.8 and 31.6%) of four species were lower than that of the IR regions (43%). The higher GC percentage in the IR regions was possibly due to the presence of four ribosomal RNA (rRNA) sequences in these regions.

The genome of four plants contained 85 protein-coding, 37 tRNA and eight rRNA genes (Table 1 and Table 2). In 85 protein-coding genes, *rps12*, *rps7*, *ndhB*, *ycf2*, *rpl23*, *rpl2* genes were repeated once. Of all these 130 predicted functional genes, 113 genes were unique, including 79 protein-coding, 30 tRNA genes and four rRNA genes (Figure 1, Table 1 and Table 2). 62 protein-coding and 22 tRNA genes were included in LSC region, whereas SSC region comprised 12 protein-coding and one tRNA genes, *ycf1* gene was located in the SSC and IRb region. Meanwhile, nine protein-coding and seven tRNA genes were repeated in the IR regions. In addition, in all these 130 genes, 51 fragments were related to self-replication (four in rRNA and 21 in tRNA), 12 genes related to small subunits of ribosome, nine genes related to large subunits of ribosome and four genes related to DNA-dependent in RNA polymerase subunits, *infA* gene were related to translational initiation factor. In addition, 46 genes were associated with photosynthesis, including six genes associated with ATP synthase, 11 with NADH oxidoreductase, six with subunits of cytochrome, seven with subunits of photosystem I, and one encoding the *rbcL* gene. In addition, there were five other genes: *matk* encoding Maturase, *cemA* encoding envelope membrane protein, *accD* encoding subunit of acetyl-CoA, *ccsA* encoding C-type cytochrome synthesis gene, and *clpP* encoding Protease (Table 2).

### 3.2. Comparisons of cp DNA of four Notopterygium Species

**Figure 2:**
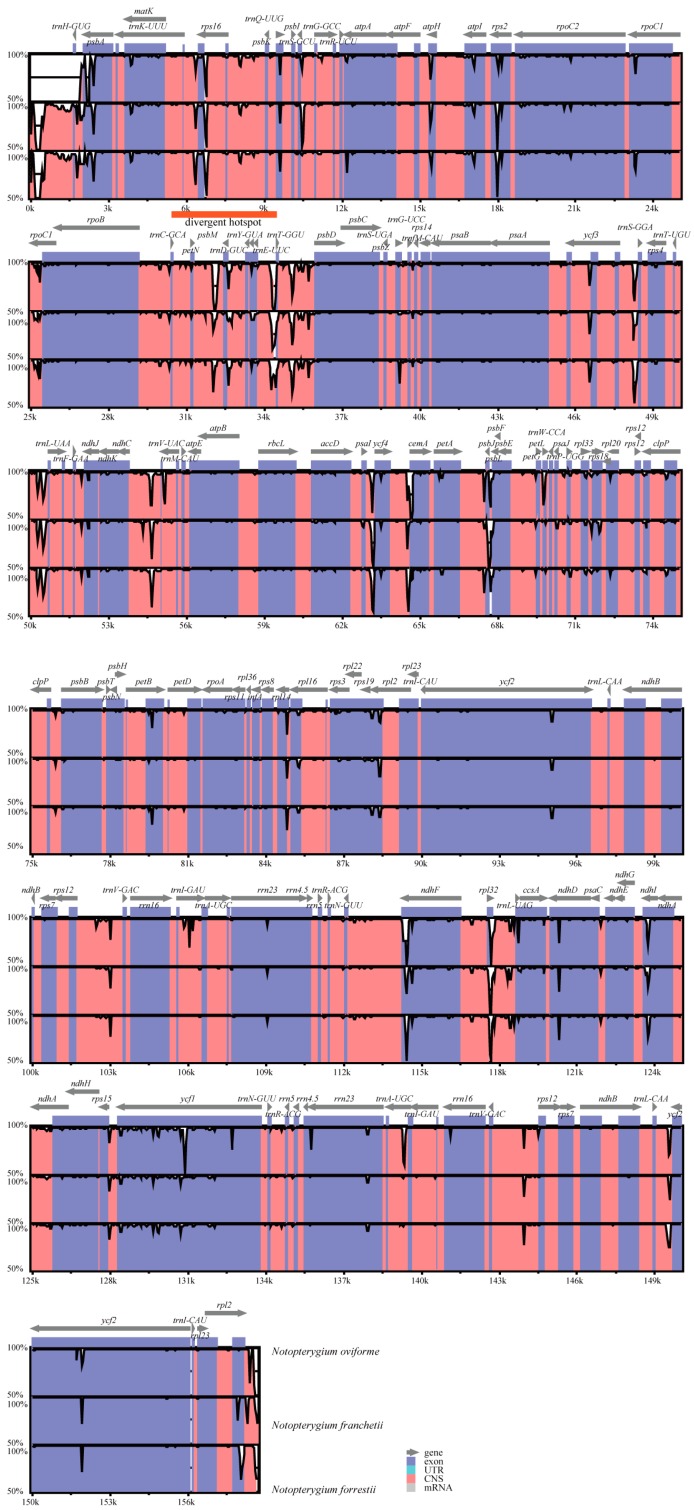
Sequence alignment of chloroplast (cp) genomes of four *Notopterygium* species. VISTA-based identity plots showing identity between four *Notopterygium* species cp genomes, with *N. incisum* as a reference. Genome regions are color coded as protein coding, rRNA coding, tRNA coding or conserved noncoding sequences. The vertical scale indicates the percentage identity, ranging from 50% to 100%. Divergent hotspot refers to the places with more variable sites compared to other regions.

**Figure 3:**
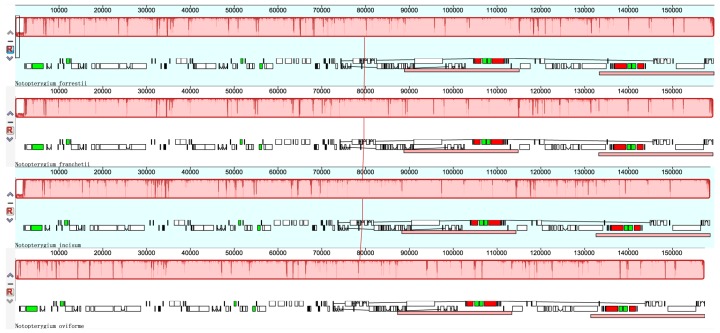
MAUVE alignment of four *Notopterygium* species chloroplast genomes. The *N. forrestii* genome is shown at top as the reference genome. Within each of the alignments, local collinear blocks are represented by blocks of the same color connected by lines.

The cp genomes of four *Notopterygium* species were relatively conserved (Figure 2), and no rearrangement occurred in gene organization after verification (Figure 3). The cp DNA size of *N. incisum* was 158,684 bp in length, and the length of *N. oviforme* was shorter than *N. incisum*, being 157,462 bp in length. The cp genome size of *N. franchetii* and *N. forrestii* were 159,389 bp, 159,607 bp respectively. Additionally, the lengths of the LSC varied in four species, from 87,304 bp of *N. oviforme* to 88,870 bp of *N. franchetii* (Table 1). In this region, 62 protein-coding and 22 tRNA genes were included; a complete *rps19* gene resided in the LSC of *N. oviforme*, while it has varying lengths in the LSC/IRb junctions among different species. (e.g., 55 bp, 46 bp and 154 bp in *N. incisum*, *N. franchetii* and *N. forrestii* respectively). Furthermore, *rps19* gene was located between the edges of LSC and IRb regions in *N. incisum*, *N. franchetii* and *N. forrestii.* However, in *N. oviforme* cp genome, *rps19* gene was completely located in LSC region and had an 89 bp distance to the edges of LSC. Meanwhile, the lengths of the SSC ranged from 17,996 bp of *N*. *oviforme* to 18290 bp of *N. franchetii*. It comprised 12 protein-coding and one tRNA gene, *ycf1* gene across SSC and IRb region. The minimum (26,081 bp) and maximum length (26,262 bp) of IR regions were located in *N. oviforme* and *N. franchetii* respectively (Table 1). Nine protein-coding and seven tRNA genes were repeated in the IR regions. Compared with other three species, the GC content (37.90%) of *N. oviforme* cp genome was slightly higher (37.90% > 37.70%) (Table 1 and Table 3).

**Figure 4:**
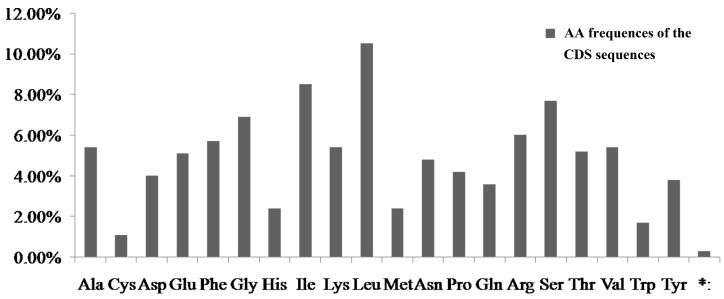
Amino acid frequencies of the *N. incisum* protein-coding sequences. * Termination codon.

Taking the example of *N. incisum*, protein-coding, rRNA, and tRNA genes were encoded by 49.50%, 5.70%, and 1.80% of the whole cp genome, respectively (Table S4). The 30 unique tRNA genes encoded all of the 20 amino acids (aa) essential for protein biosynthesis. The length of protein-coding sequences was 78,531 bp and comprised 79 unique protein genes. These protein-coding sequences coded for 26,177 codons (Table S4). Interestingly, leucine (10.50%) and cysteine (1.10%) were the maximum and minimum commonly code aa, respectively (Table S5, Figure 4). Among these codons, the maximum codon usage was TTA (852) encoding leucine, and minimum codon usage was TGC (68) encoding cysteine, respectively (Table S6).

### 3.3. Repetitive Sequences

**Figure 5:**
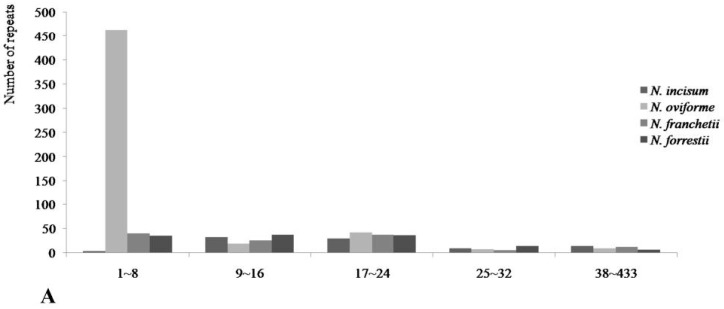

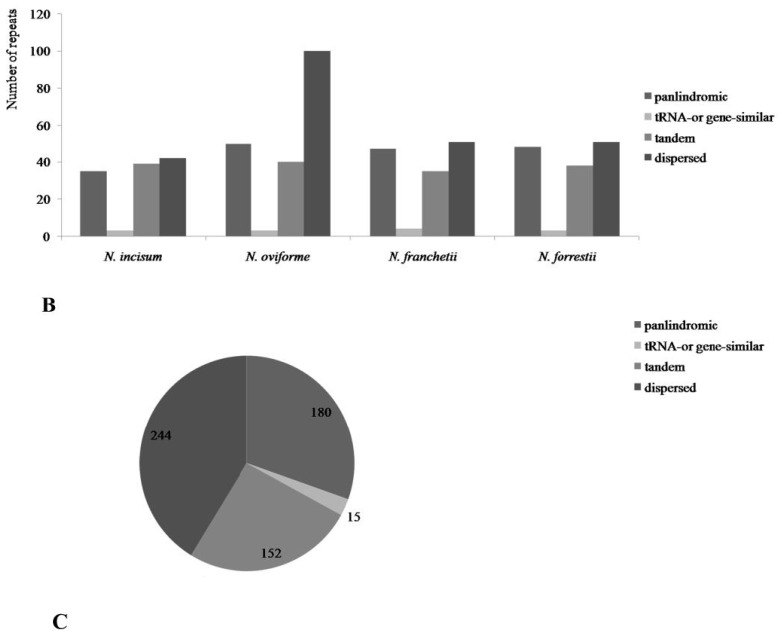
Repeat analyses. (**A**) Histogram showing the number of repeats in the four *Notopterygium* chloroplast genomes. (**B**) Compositions of the repeats from four *Notopterygium* species. (**C**) Pie chart showing the numbers of four repeat types.

The larger repeats constituted by IRa and IRb resulted in plastid genomes which contained lots of repeated sequences. In the present study, we counted four kinds of repetitions: dispersed, tandem, palindromic and tRNA or gene-similar repeats. All of the repeats were manually verified and redundant parts were removed. Among these types, the numbers and distributions of the repeats in the four cp DNA were similar and conserved (Figure 5). Dispersed unit was the most repeated type (241), the next was palindromic repeat (180), the tandem type was 152, the least one was tRNA or gene-similar repeats (15) (Figure 5). The lengths of the repeat units were mainly concentrated on 2 to 32 bp. Most of them were distributed in intergenic or intron regions, and only a minority were located in gene regions, e.g., *ycf3*, *rps18*, *ycf2, rpoC1*, *psbT*, *petB*, *ycf1*, *rps19* (Table S7). *N. oviforme* had the largest dispersed (100), palindromic (50), and tandem (40) repeats in all four species. *N. franchetii* and *N. forrestii* had similar palindromic and tandem repeats. Meanwhile, the repeats among four *Notopterygium* species had few differences; the length of repeat was mainly located in 9–24 bp region, but *N. oviforme* had larger change compared with other three species (Figure 5). In four types, dispersed, accounting for 41.2% of total repeats, were the most common (Figure 5). The largest dispersed was 463 bp in the interval of 1–8 bp repeat length and the least repeat was one bp in *N. oviforme* (Figure 5).

### 3.4. Sequence Divergence among Notopterygium Species

**Figure 6:**
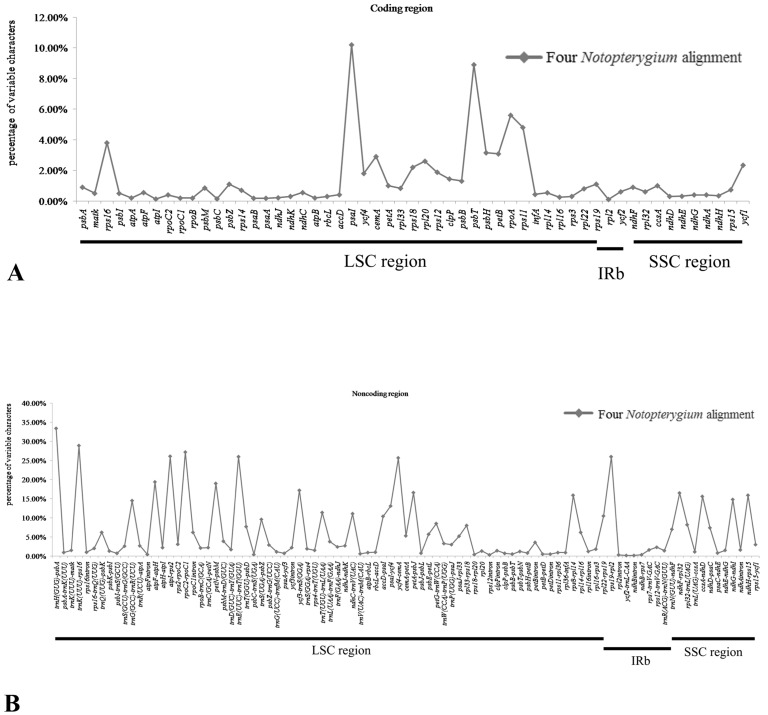
Percentages of variable characters in homologous regions among chloroplast genomes of four *Notopterygium* species. (**A**) Coding region. (**B**) Noncoding region. The homologous regions are oriented according to their locations in the chloroplast genome.

mVISTA was used to perform a sequence identity analysis, with *N. incisum* as a reference (Figure 2). The results indicated that the high sequence similarities across four cp genomes, which suggested that there were few variations, the plant evolution of *Notopterygium* was relatively conservative. In addition, as we expected, noncoding regions exhibited a higher level of divergence than coding regions in the complete cp genome. In noncoding regions, the percentage of variations ranged from 0 to 33.40%, with an average of 6.39%, which was 4.6-fold higher than that in the coding regions (1.37% on average) (Figure 6). The mean percentages of variations were 6.42%, 4% and 8% in the LSC, IRs and SSC regions in noncoding regions, respectively. There were no significant differences among these regions (1.57%, 0.35% and 0.73% for LSC, IRs and SSC regions) in the coding regions (Table S8) identified for the four species. In addition, we found that the IR regions had fewer mutations and were highly conserved in the sampled four *Notopterygium* species. More remarkably, some genes that were located in LSC region (*rps16*, *psaI*, *psbT*, *psbH*, *petB*, *rpoA* and *rps11*) exhibited higher variability (average value > 3%) than other genes. Particularly, *pasI* (10.20%) gene had a highly variable (Table S9) in the genus *Notopterygium*.

### 3.5. Evolutionary Rates of Notopterygium

**Figure 7:**
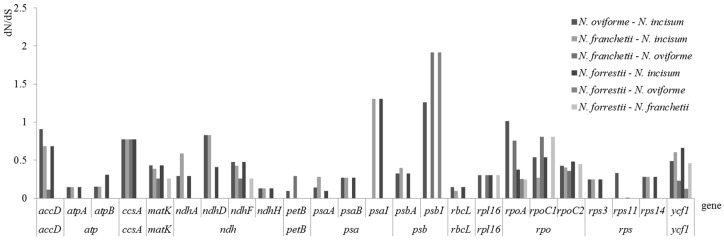
Nonsynonymous substitution (dN), Synonymous substitution (dS), and dN/dS (ω) values for individual genes or gene groups.

To estimate selection pressures of chloroplast genes, dN, dS and ω of 79 protein-coding genes were computed and compared in four *Notopterygium* species (Figure 7). Only 24 protein-coding gene have ω values from 86 pairwise comparison numerations. The ω value of the remaining comparisons could not be calculated because of dN or dS = 0. When the ω value was within 0.8–1, it contained *accD*, *ndhD*, *rpoC1* genes. The ω value of three genes (*psaI*, *psbI* and *rpoA*) significantly exceeded 1.0 (Figure 7) in four species. In a pair of *N. oviforme* and *N. incisum* species, the ω value of *psbI* and *rpoA* genes were just be seen to exceed 1. Meanwhile, the *psbI* and *rpoC1* genes exhibited the maximum dN (0.1882) and the minimum dS (0.0011) respectively (Table S10). The *psb* and *psa* genes were positively selected to a greater extent than the other genes (Figure 7). A comparison among individual genes in each functional group showed that substitution rates fluctuated widely among the 24 coding genes, with dN values ranging from 0.0006 to 0.1882 and dS values ranging from 0.0011 to 0.1495 (Table S10), respectively. Most genes exhibited ω less than 0.5, indicating the efficiency of purifying selection. Three genes (*psaI*, *psbI* and *rpoA*) were functionally essential in photosynthesis and self-replication (Table 2), that ω values were significantly greater than unity (1.3043, 1.9149 and 1.0121) in the four sampled *Notopterygium* species. It is suggested these genes were possibly under positive selection in these endangered species.

### 3.6. Phylogenomic Relationship of Notopterygium Species

**Figure 8:**
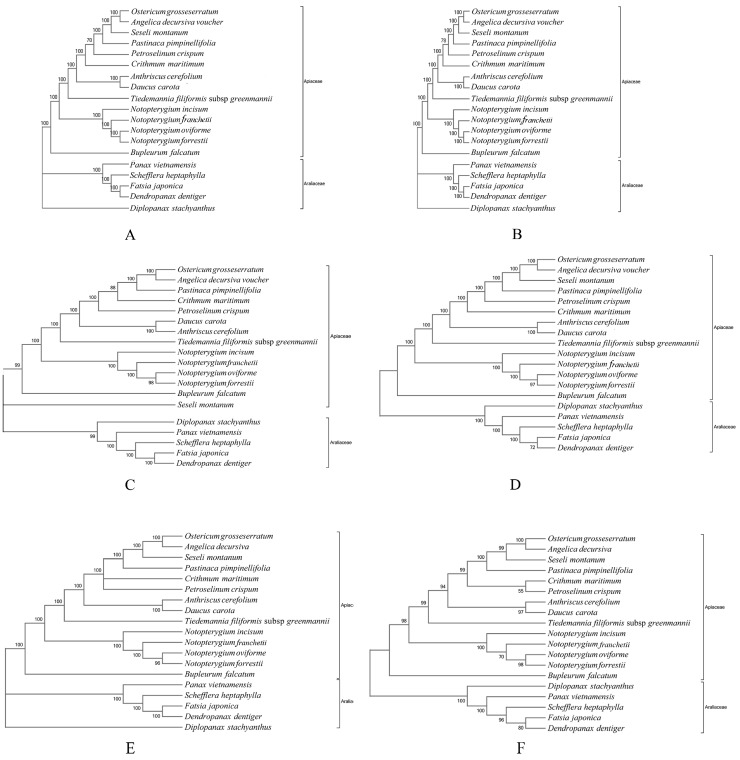
Cladogram of 19 Apiaceae and Araliaceae species using Maximum likelihood (ML) analysis based on (**A**) whole chloroplast genomes, (**B**) all data excluding an IR region, (**C**) the LSC region, (**D**) the SSC region, (**E**) the protein-coding sequences and (**F**) the IR region.

**Figure 9:**
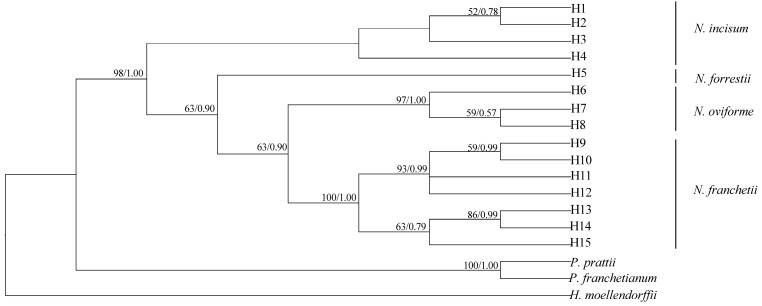
Phylogenetic relationship of ITS haplotypes of four *Notopterygium* species inferred from ML and Bayesian inference (BI) analyses. Numbers above the lines on the left indicate the ML bootstrap of clade >50%, numbers above the lines on the right indicate the Bayesian posterior probabilities.

Six data partitions (whole cp genomes; all data excluding the IR region; the LSC region; the SSC region; the protein-coding sequences and the IR region) of 19 Apiaceae and Araliaceae cp genomes were used to construct the phylogenetic relationships. The maximum likelihood (ML) analyses yielded similar topology trees in all datasets (Figure 8). The two genetic clusters were recovered, one was Apiaceae clade, another clade was corresponded to the Araliaceae species. Four *Notopterygium* species were gathered within a clade with strong bootstrap support in the cp DNA tree. In addition, the monophyletic clade of the four *Notopterygium* species was also identified by nuclear ITS datasets with strong posterior support. However, there are some inconsistent phylogenetic relationships among the four *Notopterygium* species for two types of datasets. In the cp DNA tree, *N. oviforme* and *N. forrestii* had the closer relationship, while *N. incisum* and *N. franchetii* were polyphyletic. In the ITS tree, the ITS haplotypes of each *Notopterygium* species clustered into one individual species that corresponded to themselves, respectively (Figure 9). In addition, the ITS haplotypes of *N. oviforme* and *N. franchetii* formed a large clade with moderate bootstrap.

## 4. Discussion

### 4.1. The Relationship of AT Content and Codon Usage of the Four Notopterygium Species

Many complete cp genomes of Apiaceae species were reported in NCBI, but no published cp genomes were documented for the endangered *Notopterygium* species. In the present study, we firstly obtained whole plastid genomes of four *Notopterygium* species, which will generally contribute to covering the gap in the knowledge of plastid genome evolution in *Notopterygium.* In terms of GC content, the cp genomes of *N. incisum*, *N. franchetii* and *N. forrestii* had the same GC content (37.70%), but *N. oviforme* had a subtle difference (37.90%) compared with others. The GC contents of the LSC and SSC regions (35.8% and 31.6%) were lower than that in the IR regions (43%). The high GC percentages in the IR regions was possibly due to the presence of four rRNA sequences in these regions, e.g., *rrn16*, *rrn23*, *rrn4.5*, *rrn5*. These results are consistent with the previously reported high GC percentage in the IR regions in the many complete cp genomes of angiosperms [53].

Meanwhile, the AT contents of unique 113 predicted genes were very high in the four *Notopterygium* species (Table S11). Of these genes, 79 protein-coding, 30 tRNA and four rRNA genes were included (Figure 1, Table 1 and Table 2). The 30 unique tRNA genes included all of the 20 aa required for protein biosynthesis, that also have determined the codon usage [54]. Generally speaking, composition bias was discovered as a predominant factor influencing codon bias in cp genome of plants. The composition bias of a high AT content was more matches in the noncoding regions in plants [55]. In this study, we identified the 30 tRNA genes that all had a high AT content (Table S11). Therefore, the composition bias was very similar among these genomes. In fact, two factors, with different relative importance, contributed to codon bias in different plant species [56,57]. The first reason was that the genome composition bias generated a bias in degenerated positions of coding sequences [58]. The second one was the choice of specific codons in the coding regions, most likely to increase the translation efficiency [54,56,59]. The evidence for this type of selection was primarily supported by *Escherichia coli* where codon usage was adapted to the number of tRNA in the cell [54], and the degree of codon bias varied in these genes, associated with expression level of some particular genes [56,57].

### 4.2. Sequence Divergence

In our sequenced genomes, divergent regions were often found associated with many repeats. We identified the similar high frequencies of repeats in these four cp genomes. The repeats in these species usually were located in the same genes and/or intergenic regions (e.g., *ycf2*, *ycf3*). Generally speaking, the elements of repeats were related to the rearrangement of the plastome, generating highly divergent regions via illegitimate, recombination and slipped-strand mispairing [60,61,62]. In addition, the gene order of grass cp genomes was distinct from that of standard angiosperm ones due to three typical inversions, inversion 1 (28 kb in length), inversion 2 (≈6 kb) and inversion 3 (individual *trnT* gene) [63]. A recent study of plastome from basal asterids indicated the conservation of the repeat patterns in the coding region, whereas the repeat evolution in the noncoding region was lineage-specific [64]. In the present study, we identified some repeats that were associated with two tRNA (e.g., *trnI-GAU*) copies, or gene duplication (e.g., *ycf2*/*ycf3*). These repeats might be due to similarity of gene functions and thus we classified them into another type tRNA or gene similarity [52].

Meanwhile, the genetic divergence was very low among the four *Notopterygium* cp genomes. After the four cp genomes were aligned, we plotted sequence identity using mVISTA with *N. incisum* as a reference (Figure 2). The results showed high similarities among cp genomes, with only a few sequence identities falling below 90%, suggesting that the *Notopterygium* cp genomes were rather conserved. A small divergence was found in LSC, with less mutations in IRs. In addition, one divergent hotspot region associated with a tRNA cluster in the noncoding regions containing the *trnC-GCA*, *trnD-GUC*, *trnY-GUA*, *trnT-GGU*, and *trnE-UUC* was identified in the four *Notopterygium* species. These hotspot regions identified could be used in phylogenetic analyses, or serve as potential DNA molecular barcodes [52,65,66].

### 4.3. Phylogenomic Relationships among the Four Notopterygium Species

Cp genomes of plant species provide rich sources of phylogenetic information. Numerous phylogenetic studies based on the cp DNA sequences have been carried out during the past two decades, greatly enhancing our understanding of the evolutionary relationships among angiosperms [9,67,68]. Plastid genome also has been proven to be effective in resolving difficult phylogenetic relationships among closely related species [69,70]. Complete sequencing and mutual comparative analyses demonstrated that the four *Notopterygium* cp genomes bore a high level of conservation in terms of architecture and linear sequence order. As shown in the phylogenetic tree (Figure 8), two major genetic clusters were identified, one was Apiaceae, including the sampled four *Notopterygium* species. Another one was Araliaceae clade, including five cp genomes. The monophyletic clade of the four *Notopterygium* species was identified with strong bootstrap support. In addition, *N. oviforme* and *N. forrestii* had the closely relationship in the chloroplast phylogenetic tree, however *N. oviforme* and *N. franchetii* had the closely relationship in the nrDNA ITS tree. So, there are some differences in phylogenetic relationships within the genus *Notopterygium.* We analyze the reasons for this incongruence between nuclear and chloroplast DNA phylogenies in the following. First, the nuclear ITS region is shorter and has a higher mutation rate than the chloroplast DNA (ITS: 8.85%, 54 variable sites out of 610 bp; complete chloroplast genome: 1.37%, coding region, 6.39%, noncoding region) (Tables S8 and S12). Secondly, the nuclear ITS region is biparentally inherited and dispersed by both pollen and seeds, while the chloroplast DNA fragments are maternally inherited and dispersed only by seeds [71]. Thus, the ITS region has higher intraspecific gene flow and higher efficiency of species identification than the chloroplast regions [72]. Thirdly, we did not rule out that the incongruence between nuclear and cp DNA relationships may be due to the difference of sampling strategies between two types of sequences. In this study, we sampled multiple individuals for ITS analyses, and only one individual for each cp DNA genome. In addition, the incomplete lineage sorting, convergence evolution or hybridization and gene infiltration among species [73,74,75,76,77] may have caused the incongruence between different inherited background markers. In the other words, these results together suggested that the *Notopterygium* species may have experienced a complex evolutionary history and speciation process. The present study also illustrated that the comparative analyses between cp DNA and ITS regions can help understand the comprehensive and deeply evolutionary process and phylogenetic relationships.

### 4.4. Evolutionary Rates among Notopterygium Species

A previous study showed that the alpine plants have various adaptive strategies under unpredictable environmental conditions [78]. Faster evolution of the cold-related genes had clearly been demonstrated in the evolution of alpine species [79]. For instance, the whole transcriptome analysis of *Cardamine kokaiensis* suggested that the adaptive evolution of this species to the extreme environments, particularly the chilling temperatures [78,80]. Generally speaking, cold temperatures and high irradiation were found to be not beneficial to efficient photosynthesis of plant species, and therefore, a set of photosynthetic protection strategies were desired for survival and reproduction in high altitude plants [81,82,83,84,85]. The *Notopterygium* species are mainly distributed in the alpine mountains of the Qinghai-Tibetan Plateau and adjacent regions [3]. So, these species might also have some mechanisms to adapt to cold alpine conditions. As shown above, the dN and dS rates might indicate the constraints of natural selection on organisms, estimation of these mutations (ω) played a pivotal role in understanding the dynamics of molecular evolution of plant species [86]. In this study, we identified the highest ω value was 1.9149 for *psbI* gene, far exceeding one among the four *Notopterygium* species. The other two genes were *psaI* (1.3043) and *rpoA* (1.0121), whose ω values both exceeded one (Table S10). Noticeably, *rpoA* and *psaI* genes had also been found to have significant variance in coding regions (Table S9). These genes might have suffered positive selection in these endangered *Notopterygium* species. For the pair of *N. oviforme* and *N. incisum*, the ω value of the *accD*, *ccsA* and *ndhD* genes were approximately one. The estimations of substitution rate of these genes indicated that *N. incisum* and *N. oviforme* had evolved at variable rates, and patterns of substitution were differentiated in functional categories and lineages. Based on our knowledge of the genomic information, these variable rates were related to a number of factors, such as functional constraints, relaxed or positive selection, and gene expression level [87].

Similarly, *psaI* and *psbI*, encoding photosystems subunits (Table 2), played an important role in the life history of plant. In addition, the *rpoA* gene encoding DNA-dependent RNA polymerase (Table 2) and the α subunit of RNA polymerase (RNAP) in plants [87]. The *rpoA* gene was also observed in the cp of live moss protonemata cells. These results indicated that the strong selective pressure may have acted against broad variations in these functional genes [88]. In this study, we identified that the *psbI* gene has great differences between the four *Notopterygium* species. Compared with *N. incisum*, *psbI* gene contracted in size and shifted left in location in the *N. oviforme* cp genome. Extensively, the mutation rates were more variable in the genes that contained *accD*, *ccsA*, *matK*, *ndhD*, *psaI*, *psbI*, and *ycf1* genes, as evidenced by the higher ω value (Figure 7). Here the other two genes, *ycf1* and *accD* genes had been reported in other plant lineages and had also experienced fast evolution. For instance, the *ycf1* gene had been classified as the most divergent one in plastome of vascular plants [89]. The *accD* had been proven to be an essential gene in the cp genome evolution [90]. More significantly, there was direct evidence that the *accD* gene could affect plant fitness and leaf longevity [91]. Consequently, these results indicated that these genes might be involved in the adaptation to the specific ecological environment during the evolution of *Notopterygium* species.

## Figures and Tables

**Table 1:** The features of chloroplast genomes of four *Notopterygium* species.

Species	Size (bp)	LSC (bp)	SSC (bp)	IR (bp)	Number of Protein-Coding Genes ^a^	Number of tRNA Genes ^a^	Number of rRNA Genes ^a^	GC Content (%)
*N. incisum*	158,684	88,260	18,232	26,096	85 (6)	37 (7)	8 (4)	37.70
*N. oviforme*	157,462	87,304	17,996	26,081	85 (6)	37 (7)	8 (4)	37.90
*N. franchetii*	159,389	88,749	18,290	26,175	85 (6)	37 (7)	8 (4)	37.70
*N. forrestii*	159,607	88,870	18,212	26,262	85 (6)	37 (7)	8 (4)	37.70

**^a^** The numbers in parenthesis indicate the genes duplicated in the IR regions. LSC: large single-copy; SSC: small single copy; IR: inverted repeats; tRNA: transfer RNA; rRNA: ribosomal RNA.

**Table 2:** List of genes present in *Notopterygium* chloroplast genomes.

Category	Gene Group	Gene Name				
Self-replication	Ribosomal RNA genes	*rrn16*	*rrn23*	*rrn4.5*	*rrn5*	
	Transfer RNA genes	*trnI-CAU*(2)	*trnI-GAU*(2)	*trnL-UAA*	*trnL-CAA*(2)	*trnL-UAG*
		*trnR-UCU*	*trnR-ACG*(2)	*trnA-UGC*(2)	*trnW-CCA*	*trnM-CAU*
		*trnV-UAC*	*trnV-GAC*(2)	*trnF-GAA*	*trnT-UGU*	*trnT-GGU*
		*trnP-UGG*	*trnfM-CAU*	*trnG-UCC*	*trnG-GCC*	*trnS-GGA*
		*trnS-UGA*	*trnS-GCU*	*trnD-GUC*	*trnC-GCA*	*trnN-GUU*(2)
		*trnE-UUC*	*trnY-GUA*	*trnQ-UUG*	*trnK-UUU*	*trnH-GUG*
	Small subunit of ribosome	*rps2*	*rps3*	*rps4*	*rps7*	*rps8*
		*rps11*	*rps12*	*rps14*	*rps15*	*rps16*
		*rps18*	*rps19*			
	Large subunit of ribosome	*rp12*	*rp114*	*rp116*	*rp120*	*rp122*
		*rp123*	*rp132*	*rp133*	*rp136*	
	DNA-dependent RNA polymerase	*rpoA*	*rpoB*	*rpoC1*	*rpoC2*	
	Translational initiation factor	*infA*				
Genes for photosynthesis	Subunits of photosystem I	*psaA*	*psaB*	*psaC*	*psaI*	*psaJ*
		*ycf3*	*ycf4*			
	Subunits of photosystem II	*psbA*	*psbB*	*psbC*	*psbD*	*psbE*
		*psbF*	*psbH*	*psbI*	*psbJ*	*psbK*
		*psbL*	*psbM*	*psbN*	*psbT*	*psbZ*
	NADH oxidoreductase	*ndhA*	*ndhB*	*ndhC*	*ndhD*	*ndhE*
		*ndhF*	*ndhG*	*ndhH*	*ndhI*	*ndhJ*
		*ndhK*				
	Subunits of cytochrome	*petA*	*petB*	*petD*	*petG*	*petL*
		*petN*				
	Subunits of ATP	*atpA*	*atpB*	*atpE*	*atpF*	*atpH*
	synthase	*atpI*				
	Large subunit of Rubisco	*rbcL*				
Other genes	Maturase	*matk*				
	Envelope membrane protein	*cemA*				
	Subunit of acetyl-CoA	*accD*				
	C-type cytochrome synthesis gene	*ccsA*				
	Protease	*clpP*				
Conserved reading frames	Conserved Open Reading Frames	*ycf1*	*ycf2*			

**Table 3:** Base compositions of chloroplast genomes of four *Notopterygium* species.

Region		*N. incisum*	*N. oviforme*	*N. franchetii*	*N. forrestii*
LSC (%)	A	31.5	31.4	31.5	31.6
	T	32.7	32.6	32.7	32.6
	C	18.4	18.3	18.4	18.4
	G	17.4	17.7	17.4	17.4
	GC	35.8	36.0	35.8	35.8
SSC (%)	A	34.6	34.4	34.6	34.6
	T	33.8	33.8	33.8	33.8
	C	16.5	16.5	16.5	16.4
	G	15.1	15.2	15.1	15.1
	GC	31.6	31.7	31.6	31.6
IRa (%)	A	28.3	28.2	28.3	28.3
	T	28.7	28.5	28.7	28.7
	C	22.1	22.1	22.2	22.2
	G	20.8	21.2	20.8	20.8
	GC	42.9	43.3	43.0	43.0
IRb (%)	A	28.7	28.6	28.7	28.6
	T	28.3	28.2	28.3	28.4
	C	20.8	21.0	20.8	20.8
	G	22.1	22.2	22.2	22.2
	GC	42.9	43.2	43.0	43.0
overall length (%)	A	30.9	30.8	30.9	30.9
	T	31.4	31.3	31.4	31.4
	C	19.2	19.2	19.2	19.2
	G	18.5	18.8	18.5	18.5
	GC	37.7	37.9	37.7	37.7

## Supplementary Materials

The following are available online at www.mdpi.com/2073-4425/8/4/124/s1. Table S1: Sample information of cp DNA and ITS datasets of four *Notopterygium* species, Table S2: Ten complete chloroplast genomes of Apiaceae and five complete chloroplast genomes of Araliaceae species from GenBank, Table S3: Assembled reads of four *Notopterygium* species, Table S4: The encode frequencies of protein-coding, tRNA and rRNA sequences of *Notopterygium incisum*, Table S5: Amino acid frequencies of chloroplast protein-coding sequences of *Notopterygium*, Table S6: Codon and the frequencies of amino acid in *Notopterygium incisum*, Table S7: Repeat sequences in *Notopterygium* chloroplast genomes (see details in the excel file entitled Table S7), Table S8: The variation rates in *Notopterygium* chloroplast genomes, Table S9: Percentages of variable characters in coding and noncoding regions, Table S10: Nonsynonymous substitution (dN), Synonymous substitution (dS), and dN/dS(ω) values for individual genes or gene groups (see details in the excel file entitled Table S10), Table S11: The AT contents of gene in coding and noncoding regions, Table S12: The variable sites of ITS sequence of *Notopterygium* species (see details in the excel file entitled Table S12).
