# Supplementary material for: Comparative Analysis of the Complete Chloroplast Genome of Four Endangered Herbals of Notopterygium
Source: Genes (Basel). 2017 Apr 19;8(4):124. doi: 10.3390/genes8040124 (PMC5406871; doi:10.3390/genes8040124)
Supplement: Supplementary file 1 [file genes-08-00124-s001.zip › genes-185530-supplementary-last/genes-185530-supplementary(1)/Supporting information.docx]

Table S1: Sample information of cp DNA and ITS datasets of four *Notopterygium* species.

Table S2: Ten complete chloroplast genomes of Apiaceae and five complete chloroplast genomes of Araliaceae species from GenBank.

Table S3: Assembled reads of four *Notopterygium* species.

Table S4: The encode frequencies of protein-coding, tRNA and rRNA sequences of *Notopterygium incisum.*

Table S5: Amino acid frequencies of chloroplast protein-coding sequences of *Notopterygium*.

Table S6: Codon and the frequencies of amino acid in *Notopterygium incisum*.

Table S7: Repeat sequences in *Notopterygium* chloroplast genomes (see details in the excel file entitled Table S7).

Table S8: The variation rates in *Notopterygium* chloroplast genomes.

Table S9: Percentages of variable characters in coding and noncoding regions.

Table S10: Nonsynonymous substitution (dN), Synonymous substitution (dS), and dN/dS(ω) values for individual genes or gene groups (see details in the excel file entitled Table S10).

Table S11: The AT contents of gene in coding and noncoding regions.

Table S12: The variable sites of ITS sequence of *Notopterygium* species (see details in the excel file entitled Table S12).

**Table S1:** Sample information of cp DNA and ITS datasets of four *Notopterygium* species.

| Code. | Species | Sample sites | Longituide | Latitude | Elevation/m | Cp DNA  (Number) | ITS  (Number and ITS haplotype) |
| --- | --- | --- | --- | --- | --- | --- | --- |
| E | *N. incisum* | Changan area, Shaanxi | 107.701 | 34.0535 | 3190 | 1 | 6 (H1-H4) |
| F | *N. incisum* | Datong,Qinghai | 101.4009 | 37.1007 | 2756 |  | 10 |
| LQC | *N. forrestii* | Litang,Sichuan | 100.3092 | 29.9981 | 4010 | 1 | 10 (H5) |
| LQA | *N. forrestii* | Yajiang,Sichuan | 100.5662 | 30.1583 | 4164 |  | 10 |
| LQ | *N. oviforme* | Fengyukou, Shaanxi | 108.623 | 33.6205 | 1890 | 1 | 5 (H6-H8) |
| LA | *N. oviforme* | Taibai,Shaanxi | 107.7011 | 34.0535 | 3190 |  | 10 |
| KK | *N. franchetii* | Nuogaier, Sichuan | 102.9615 | 33.5903 | 3526 | 1 | 10 (H9-H12) |
| KG | *N. franchetii* | Yaan,Sichuan | 102.8176 | 30.3683 | 2890 |  | 10 (H13-H15) |
| XX | *P. prattii* | Yajiang, Sichuan | 100.7859 | 30.0441 | 4220 |  | 1 |
| YY | *P. franchetianum* | Xiaojin, Sichuan | 102.8827 | 30.9478 | 4050 |  | 1 |
| DH | *H. moellendorffii* | Jiexiu mountain, Shanxi | 111.9883 | 36.8954 | 1944 |  | 1 |

Note: For ITS analyses, genomic DNA of 71 individuals from four *Notopterygium* species were extracted using a modified CTAB protocol [1],We selected ITS4 and ITS5 (F:TCCTTCCGCTTATTGATATGC; R: GGAAGGAGAAGTCGTAACAAGG) primer for amplifying the entire internal transcribed spacer (ITS) region [2-4]. The detailed conditions of PCR were as follows: an initial denaturation step of 5 min at 94°C, followed by 36 cycles of 40 sec at 94°C denaturation, 40 sec at 52°C annealing and 90 sec at 72°C extension, and a final extension at 72°C for 10 min. The PCR products were purified and sequenced by Sangon Biotechnology (Shanghai, China). DNA sequences were aligned with BIOEDIT v7.0.9 [5]. The number of haplotypes were estimated by DnaSP v5.00.04 [6]. All sequences generated in this study have been deposited into GenBank under the accession numbers KY848833-KY848847.

References:

1. Doyle, J.J. A rapid DNA isolation procedure for small quantities of fresh leaf tissue. *Phytochem. bull***1987**, 19, 11-15.
2. Viega, J.; Bloch, J. T.; Kohno, Y.; McGraw, G. ITS4: A static vulnerability scanner for C and C++ code. In *Computer Security Applications, ACSAC'00. 16th Annual Conference* **2000**, 257-267.
3. Mitchell, T. G.; Freedman, E. Z.; White, T. J.; Taylor, J. W. Unique oligonucleotide primers in PCR for identification of Cryptococcus neoformans.*J. Clin.Microbiol*. **1994**, 32, 253-255.
4. White, T.J.; Bruns, T.; Lee, S.; Taylor, J. “Amplification and directsequencing of fungal RNA genes for phylogenetics.” in *PCR Protocols: AGuide to Methods and Applications* ed. M.A. Innis, D.H. Gelfand, J.J. Sninsky,and T.J. White. (New York: Academic Press), **1990**,315-322.

5 Hall, T.A. BioEdit: a user-friendly biological sequence alignment editor and analysis program for Windows 95/98/NT. *Nucleic Acids Symp. Ser.* **1999**, 41, 95-98.

6 Librado, P.; and Rozas, J. DnaSP v5: a software for comprehensive analysis of813 DNA polymorphism data. *Bioinformatics* **2009**,25, 1451-1452.

**Table S2:** Ten complete chloroplast genomes of Apiaceae and five complete chloroplast genomes of Araliaceae species from GenBank.

| Species |  | GenBank accessions | |
| --- | --- | --- | --- |
| *Anthriscus cerefolium* | | | GU456628 |
| *Daucus carota* | | | DQ898156 |
| *Tiedemannia filiformis* subsp *greenmannii* | | | HM596071 |
| *Crithmum maritimum* | | | HM596072 |
| *Angelica decursiva* | | | KT781591 |
| *Ostericum grosseserratum* | | | KT852844 |
| *Bupleurum falcatum* | | | KM207676 |
| *Seseli montanum* | | | KM035851 |
| *Pastinaca pimpinellifolia* | | | KM035850 |
| *Petroselinum crispum* | | | HM596073 |
| [*Panax vietnamensis*](https://www.ncbi.nlm.nih.gov/nuccore/NC_028704.1) | | | NC_028704 |
| [*Diplopanax stachyanthus*](https://www.ncbi.nlm.nih.gov/nuccore/NC_029750.1) | | | NC_029750 |
| [*Schefflera heptaphylla*](https://www.ncbi.nlm.nih.gov/nuccore/NC_029764.1) | | | NC_029764 |
| [*Fatsia japonica*](https://www.ncbi.nlm.nih.gov/nuccore/NC_027685.1) | | | NC_027685 |
| [*Dendropanax dentiger*](https://www.ncbi.nlm.nih.gov/nuccore/NC_026546.1) | | | NC_026546 |

| Species | Assembled Reads | Coverage (Min.) | Coverage (Max.) | Coverage  (Mean) |
| --- | --- | --- | --- | --- |
| *N. incisum* | 357,226 | 5 | 1313 | 566.9 |
| *N. oviforme* | 275,964 | 5 | 666 | 265.3 |
| *N. franchetii* | 110,460 | 10 | 493 | 174.7 |
| *N. forrestii* | 151,256 | 5 | 754 | 239.4 |

**Table S3:** Assembled reads of four *Notopterygium* species.

**Table S4:** The encode frequencies of protein-coding, tRNA and rRNA sequences of *Notopterygium incisum.*

| Names | Types | Minimum | Maximum | Lengths | Encoded frequencies |
| --- | --- | --- | --- | --- | --- |
| CDS | Coding region |  |  | 78,531 | 78,531/158,684=49.5% |
| rRNA | Coding region |  |  | 9074 | 9074/158,684=5.7% |
| tRNA | Coding region |  |  | 2787 | 2787/158,684=1.8% |
|  | Noncoding region |  |  |  | 100-49.5%-5.7%-1.8%=43% |
| *rpl2* | CDS | 157,077 | 158,552 | 825 |  |
| *rpl23* | CDS | 156,777 | 157,058 | 282 |  |
| *ycf2* | CDS | 150,105 | 156,449 | 6,345 |  |
| *ndhB* | CDS | 146,533 | 148,753 | 1,533 |  |
| *rps7* | CDS | 145,767 | 146,234 | 468 |  |
| *ycf1* | CDS | 128,655 | 134,135 | 5,481 |  |
| *rps15* | CDS | 127,978 | 128,250 | 273 |  |
| *ndhH* | CDS | 126,689 | 127,870 | 1,182 |  |
| *ndhA* | CDS | 124,506 | 126,687 | 1,092 |  |
| *ndhI* | CDS | 123,909 | 124,412 | 504 |  |
| *ndhG* | CDS | 122,978 | 123,508 | 531 |  |
| *ndhE* | CDS | 122,454 | 122,777 | 324 |  |
| *psaC* | CDS | 121,942 | 122,187 | 246 |  |
| *ndhD* | CDS | 120,324 | 121,853 | 1,530 |  |
| *ccsA* | CDS | 119,163 | 120,134 | 972 |  |
| *rpl32* | CDS | 117,900 | 118,064 | 165 |  |
| *ndhF* | CDS | 114,584 | 116,827 | 2,244 |  |
| *ycf2* | CDS | 90,496 | 96,840 | 6,345 |  |
| *ndhB* | CDS | 98,192 | 100,412 | 1,533 |  |
| *rpl23* | CDS | 89,887 | 90,168 | 282 |  |
| *rpl2* | CDS | 88,393 | 89,868 | 825 |  |
| *rps7* | CDS | 100,711 | 101,178 | 468 |  |
| *rps19* | CDS | 88,037 | 88,315 | 279 |  |
| *rpl22* | CDS | 87,470 | 87,964 | 495 |  |
| *rps3* | CDS | 86,832 | 87,485 | 654 |  |
| *rpl16* | CDS | 85,306 | 86,665 | 408 |  |
| *rpl14* | CDS | 84,800 | 85,168 | 369 |  |
| *rps8* | CDS | 84,188 | 84,592 | 405 |  |
| *infA* | CDS | 83,833 | 84,066 | 234 |  |
| *rpl36* | CDS | 83,603 | 83,716 | 114 |  |
| *rps11* | CDS | 83,069 | 83,485 | 417 |  |
| *rpoA* | CDS | 81,933 | 82,994 | 1,062 |  |
| *petD* | CDS | 80,551 | 81,797 | 483 |  |
| *petB* | CDS | 78,971 | 80,365 | 648 |  |
| *psbH* | CDS | 78,526 | 78,840 | 315 |  |
| *psbN* | CDS | 78,385 | 78,516 | 132 |  |
| *psbT* | CDS | 78,188 | 78,298 | 111 |  |
| *psbB* | CDS | 76,460 | 77,992 | 1,533 |  |
| *clpP* | CDS | 73,952 | 76,008 | 693 |  |
| *rps12* | CDS | 73,685 | 102,027 | 372 |  |
| *rps12* | CDS | 73,631 | 145,711 | 399 |  |
| *rpl20* | CDS | 72,530 | 72,940 | 411 |  |
| *rps18* | CDS | 71,996 | 72,301 | 306 |  |
| *rpl33* | CDS | 71,564 | 71,803 | 240 |  |
| *psaJ* | CDS | 71,025 | 71,153 | 129 |  |
| *petG* | CDS | 70,076 | 70,195 | 120 |  |
| *petL* | CDS | 69,834 | 69,929 | 96 |  |
| *psbE* | CDS | 68,382 | 68,819 | 438 |  |
| *psbF* | CDS | 68,253 | 68,372 | 120 |  |
| *psbL* | CDS | 68,114 | 68,311 | 198 |  |
| *psbJ* | CDS | 67,859 | 67,981 | 123 |  |
| *petA* | CDS | 65,874 | 66,869 | 996 |  |
| *cemA* | CDS | 64,970 | 65,659 | 690 |  |
| *ycf4* | CDS | 63,617 | 64,171 | 555 |  |
| *psaI* | CDS | 63,095 | 63,205 | 111 |  |
| *accD* | CDS | 61,144 | 62,613 | 1,470 |  |
| *rbcL* | CDS | 59,104 | 60,531 | 1,428 |  |
| *atpB* | CDS | 56,842 | 58,335 | 1,494 |  |
| *atpE* | CDS | 56,444 | 56,845 | 402 |  |
| *ndhC* | CDS | 53,725 | 54,087 | 363 |  |
| *ndhK* | CDS | 52,999 | 53,676 | 678 |  |
| *ndhJ* | CDS | 52,412 | 52,888 | 477 |  |
| *rps4* | CDS | 49,150 | 49,755 | 606 |  |
| *ycf3* | CDS | 45,991 | 48,007 | 507 |  |
| *psaA* | CDS | 43,033 | 45,285 | 2,253 |  |
| *psaB* | CDS | 40,803 | 43,007 | 2,205 |  |
| *rps14* | CDS | 40,377 | 40,679 | 303 |  |
| *psbZ* | CDS | 39,398 | 39,586 | 189 |  |
| *psbC* | CDS | 37,290 | 38,711 | 1,422 |  |
| *psbD* | CDS | 36,281 | 37,342 | 1,062 |  |
| *psbM* | CDS | 32,775 | 32,891 | 117 |  |
| *petN* | CDS | 31,488 | 31,577 | 90 |  |
| *rpoB* | CDS | 26,254 | 29,466 | 3,213 |  |
| *rpoC1* | CDS | 23,428 | 26,248 | 2,058 |  |
| *rpoC2* | CDS | 19,038 | 23,216 | 4,179 |  |
| *rps2* | CDS | 18,102 | 18,812 | 711 |  |
| *atpI* | CDS | 17,086 | 17,829 | 744 |  |
| *atpH* | CDS | 15,674 | 15,919 | 246 |  |
| *atpF* | CDS | 14,019 | 15,281 | 546 |  |
| *atpA* | CDS | 12,441 | 13,964 | 1,524 |  |
| *psbI* | CDS | 10,381 | 10,491 | 111 |  |
| *psbK* | CDS | 9,817 | 10,002 | 186 |  |
| *rps16* | CDS | 6,778 | 7,885 | 237 |  |
| *matK* | CDS | 3,940 | 5,466 | 1,527 |  |
| *psbA* | CDS | 2,360 | 3,436 | 1,077 |  |
| *rrn5* | rRNA | 111,374 | 111,494 | 121 |  |
| *rrn4.5* | rRNA | 110,983 | 111,085 | 103 |  |
| *rrn23* | rRNA | 108,063 | 110,884 | 2,822 |  |
| *rrn16* | rRNA | 104,152 | 105,642 | 1,491 |  |
| *rrn16* | rRNA | 141,303 | 142,793 | 1,491 |  |
| *rrn23* | rRNA | 136,061 | 138,882 | 2,822 |  |
| *rrn4.5* | rRNA | 135,860 | 135,962 | 103 |  |
| *rrn5* | rRNA | 135,451 | 135,571 | 121 |  |
| *trnV*(UAC) | tRNA | 55,340 | 55,986 | 74 |  |
| *trnW*(CCA) | tRNA | 70,334 | 70,407 | 74 |  |
| *trnM*(CAU) | tRNA | 56,161 | 56,233 | 73 |  |
| *trnF*(GAA) | tRNA | 51,960 | 52,032 | 73 |  |
| *trnL*(UAA) | tRNA | 51,008 | 51,593 | 85 |  |
| *trnT*(UGU) | tRNA | 50,095 | 50,170 | 76 |  |
| *trnS*(GGA) | tRNA | 48,740 | 48,826 | 87 |  |
| *trnfM*(CAU) | tRNA | 40,145 | 40,218 | 74 |  |
| *trnG*(UCC) | tRNA | 39,889 | 39,959 | 71 |  |
| *trnS*(UGA) | tRNA | 38,937 | 39,029 | 93 |  |
| *trnT*(GGU) | tRNA | 34,779 | 34,850 | 72 |  |
| *trnE*(UUC) | tRNA | 33,957 | 34,029 | 73 |  |
| *trnY*(GUA) | tRNA | 33,803 | 33,886 | 84 |  |
| *trnD(*GUC) | tRNA | 33,609 | 33,682 | 74 |  |
| *trnC*(GCA) | tRNA | 30,727 | 30,797 | 71 |  |
| *trnG*(GCC) | tRNA | 11,280 | 12,048 | 64 |  |
| *trnR*(UCU) | tRNA | 12,259 | 12,330 | 72 |  |
| *trnS*(GCU) | tRNA | 10,631 | 10,718 | 88 |  |
| *trnQ*(UUG) | tRNA | 9,394 | 9,465 | 72 |  |
| *trnK*(UUU) | tRNA | 3,630 | 6,222 | 72 |  |
| *trnH*(GUG) | tRNA | 1,959 | 2,033 | 75 |  |
| *trnP*(UGG) | tRNA | 70,556 | 70,629 | 74 |  |
| *trnR*(ACG) | tRNA | 135,119 | 135,192 | 74 |  |
| *trnA*(UGC) | tRNA | 139,035 | 139,921 | 73 |  |
| *trnI*(GAU) | tRNA | 139,986 | 141,008 | 72 |  |
| *trnV*(GAC) | tRNA | 143,021 | 143,092 | 72 |  |
| *trnI*(CAU) | tRNA | 156,538 | 156,611 | 74 |  |
| *trnL*(CAA) | tRNA | 149,328 | 149,408 | 81 |  |
| *trnN*(GUU) | tRNA | 134,463 | 134,534 | 72 |  |
| *trnL*(UAG) | tRNA | 118,996 | 119,075 | 80 |  |
| *trnV*(GAC) | tRNA | 103,853 | 103,924 | 72 |  |
| *trnL*(CAA) | tRNA | 97,537 | 97,617 | 81 |  |
| *trnI*(CAU) | tRNA | 90,334 | 90,407 | 74 |  |
| *trnR*(ACG) | tRNA | 111,753 | 111,826 | 74 |  |
| *trnA*(UGC) | tRNA | 107,024 | 107,910 | 73 |  |
| *trnI*(GAU) | tRNA | 105,937 | 106,959 | 72 |  |
| *trnN*(GUU) | tRNA | 112,411 | 112,482 | 72 |  |

CDS, coding sequences.

**Table S5:** Amino acid frequencies of chloroplast protein-coding sequences of *Notopterygium*.

| AA | Frequencies of species | | | | | | | |
| --- | --- | --- | --- | --- | --- | --- | --- | --- |
|  | *N. incisum* | % | *N. oviforme* | % | *N. franchetii* | % | *N. forrestii* | % |
| Ala | 1,405 | 5.40% | 1,381 | 5.30% | 1,384 | 5.30% | 1,408 | 5.40% |
| Cys | 287 | 1.10% | 283 | 1.10% | 281 | 1.10% | 287 | 1.10% |
| Asp | 1,054 | 4.00% | 1,054 | 4.10% | 1,059 | 4.10% | 1,064 | 4.10% |
| Glu | 1,331 | 5.10% | 1,328 | 5.10% | 1,320 | 5.10% | 1,334 | 5.10% |
| Phe | 1,498 | 5.70% | 1,477 | 5.70% | 1,482 | 5.70% | 1,488 | 5.70% |
| Gly | 1,799 | 6.90% | 1,758 | 6.80% | 1,768 | 6.80% | 1,798 | 6.90% |
| His | 627 | 2.40% | 626 | 2.40% | 620 | 2.40% | 627 | 2.40% |
| Ile | 2,212 | 8.50% | 2,204 | 8.50% | 2,190 | 8.40% | 2,218 | 8.50% |
| Lys | 1,403 | 5.40% | 1,379 | 5.30% | 1,390 | 5.40% | 1,410 | 5.40% |
| Leu | 2,760 | 10.50% | 2,731 | 10.60% | 2,739 | 10.60% | 2,762 | 10.50% |
| Met | 621 | 2.40% | 609 | 2.40% | 615 | 2.40% | 617 | 2.40% |
| Asn | 1,245 | 4.80% | 1,224 | 4.70% | 1,227 | 4.70% | 1,240 | 4.70% |
| Pro | 1,097 | 4.20% | 1,079 | 4.20% | 1,089 | 4.20% | 1,095 | 4.20% |
| Gln | 936 | 3.60% | 916 | 3.50% | 923 | 3.60% | 929 | 3.50% |
| Arg | 1,582 | 6.00% | 1,551 | 6.00% | 1,566 | 6.00% | 1,588 | 6.10% |
| Ser | 2,022 | 7.70% | 1,996 | 7.70% | 2,020 | 7.80% | 2,033 | 7.80% |
| Thr | 1,369 | 5.20% | 1,344 | 5.20% | 1,348 | 5.20% | 1,364 | 5.20% |
| Val | 1,403 | 5.40% | 1,380 | 5.30% | 1,382 | 5.30% | 1,395 | 5.30% |
| Trp | 453 | 1.70% | 448 | 1.70% | 454 | 1.80% | 453 | 1.70% |
| Tyr | 988 | 3.80% | 965 | 3.70% | 977 | 3.80% | 988 | 3.80% |
| * | 85 | 0.30% | 85 | 0.30% | 85 | 0.30% | 85 | 0.30% |

**Table S6:** Codon and the frequencies of amino acid in *Notopterygium incisum*.

| AA | Codons | % of AA | Frequencies |
| --- | --- | --- | --- |
| Ala | GCA | 28.50% | 400 |
|  | GCC | 16.90% | 238 |
|  | GCG | 10.70% | 151 |
|  | GCT | 43.80% | 616 |
| Cys | TGC | 23.70% | 68 |
|  | TGT | 76.30% | 219 |
| Asp | GAC | 19.00% | 200 |
|  | GAT | 81.00% | 854 |
| Glu | GAA | 74.20% | 987 |
|  | GAG | 25.80% | 344 |
| Phe | TTC | 35.40% | 530 |
|  | TTT | 64.60% | 968 |
| Gly | GGA | 38.40% | 690 |
|  | GGC | 11.10% | 199 |
|  | GGG | 17.60% | 317 |
|  | GGT | 33.00% | 593 |
| His | CAC | 24.90% | 156 |
|  | CAT | 75.10% | 471 |
| Ile | ATA | 32.80% | 726 |
|  | ATC | 20.80% | 459 |
|  | ATT | 46.40% | 1,027 |
| Lys | AAA | 73.50% | 1,031 |
|  | AAG | 26.50% | 372 |
| Leu | CTA | 13.90% | 383 |
|  | CTC | 7.00% | 192 |
|  | CTG | 6.40% | 176 |
|  | CTT | 21.40% | 590 |
|  | TTA | 30.90% | 852 |
|  | TTG | 20.50% | 567 |
| Met | ATA | 0.50% | 3 |
|  | ATC | 0.20% | 1 |
|  | ATG | 97.70% | 607 |
|  | ATT | 1.00% | 6 |
|  | CTG | 0.30% | 2 |
|  | GTG | 0.20% | 1 |
|  | TTG | 0.20% | 1 |
| Asn | AAC | 24.90% | 310 |
|  | AAT | 75.10% | 935 |
| Pro | CCA | 27.20% | 298 |
|  | CCC | 18.00% | 198 |
|  | CCG | 15.40% | 169 |
|  | CCT | 39.40% | 432 |
| Gln | CAA | 75.10% | 703 |
|  | CAG | 24.90% | 233 |
| Arg | AGA | 30.80% | 488 |
|  | AGG | 10.10% | 160 |
|  | CGA | 22.40% | 355 |
|  | CGC | 5.90% | 94 |
|  | CGG | 8.80% | 139 |
|  | CGT | 21.90% | 346 |
| Ser | AGC | 5.70% | 115 |
|  | AGT | 20.20% | 408 |
|  | TCA | 19.70% | 399 |
|  | TCC | 16.50% | 333 |
|  | TCG | 9.60% | 195 |
|  | TCT | 28.30% | 572 |
| Thr | ACA | 29.30% | 401 |
|  | ACC | 19.10% | 261 |
|  | ACG | 12.30% | 168 |
|  | ACT | 39.40% | 539 |
| Val | GTA | 35.50% | 498 |
|  | GTC | 12.30% | 173 |
|  | GTG | 15.10% | 212 |
|  | GTT | 37.10% | 520 |
| Trp | TGG | 100% | 453 |
| Tyr | TAC | 19.80% | 196 |
|  | TAT | 80.20% | 792 |
| * | TAA | 48.20% | 41 |
|  | TAG | 28.20% | 24 |
|  | TGA | 23.50% | 20 |

*Termination codon

**Table S8:** The variation rates in *Notopterygium* chloroplast genomes.

|  | Average variation rates /% | |
| --- | --- | --- |
| Region | Coding region | Noncoding region |
| LSC | 1.57 | 6.42 |
| IR | 0.35 | 4 |
| SSC | 0.73 | 8 |
| Overall | 1.37% | 6.39% |

**Table S9:** Percentages of variable characters in coding and noncoding regions.

| Lengths | Variation points | Variation rates | Genes | Scopes | Regions |
| --- | --- | --- | --- | --- | --- |
| 1062 | 1 | 0.90% | *psbA* | LSC | coding region |
| 1527 | 8 | 0.50% | *matk* | LSC | coding region |
| 237 | 9 | 3.80% | *rps16* | LSC | coding region |
| 208 | 1 | 0.50% | *psbI* | LSC | coding region |
| 1524 | 3 | 0.20% | *atpA* | LSC | coding region |
| 546 | 3 | 0.55% | *atpF* | LSC | coding region |
| 744 | 1 | 0.13% | *atpI* | LSC | coding region |
| 4179 | 17 | 0.40% | *rpoC2* | LSC | coding region |
| 2058 | 4 | 0.19% | *rpoC1* | LSC | coding region |
| 3213 | 6 | 0.19% | *rpoB* | LSC | coding region |
| 117 | 1 | 0.85% | *psbM* | LSC | coding region |
| 1422 | 2 | 0.14% | *psbC* | LSC | coding region |
| 189 | 2 | 1.10% | *psbZ* | LSC | coding region |
| 303 | 2 | 0.70% | *rps14* | LSC | coding region |
| 2205 | 4 | 0.18% | *psaB* | LSC | coding region |
| 2253 | 4 | 0.18% | *psaA* | LSC | coding region |
| 477 | 1 | 0.21% | *ndhJ* | LSC | coding region |
| 678 | 2 | 0.30% | *ndhK* | LSC | coding region |
| 363 | 2 | 0.55% | *ndhC* | LSC | coding region |
| 1494 | 3 | 0.20% | *atpB* | LSC | coding region |
| 1428 | 4 | 0.30% | *rbcL* | LSC | coding region |
| 1470 | 6 | 0.41% | *accD* | LSC | coding region |
| 88 | 9 | 10.20% | *psaI* | LSC | coding region |
| 556 | 1 | 1.80% | *ycf4* | LSC | coding region |
| 691 | 2 | 2.90% | *cemA* | LSC | coding region |
| 997 | 1 | 1% | *petA* | LSC | coding region |
| 240 | 2 | 0.83% | *rpl33* | LSC | coding region |
| 314 | 7 | 2.20% | *rps18* | LSC | coding region |
| 387 | 1 | 2.60% | *rpl20* | LSC | coding region |
| 372 | 7 | 1.88% | *rps12* | LSC | coding region |
| 693 | 1 | 1.44% | *clpP* | LSC | coding region |
| 1534 | 2 | 1.30% | *psbB* | LSC | coding region |
| 112 | 1 | 8.90% | *psbT* | LSC | coding region |
| 317 | 1 | 3.15% | *psbH* | LSC | coding region |
| 649 | 2 | 3.08% | *petB* | LSC | coding region |
| 1063 | 6 | 5.60% | *rpoA* | LSC | coding region |
| 417 | 2 | 4.80% | *rps11* | LSC | coding region |
| 234 | 1 | 0.43% | *infA* | LSC | coding region |
| 369 | 2 | 0.54% | *rpl14* | LSC | coding region |
| 408 | 1 | 0.25% | *rpl16* | LSC | coding region |
| 654 | 2 | 0.30% | *rps3* | LSC | coding region |
| 495 | 4 | 0.80% | *rpl22* | LSC | coding region |
| 279 | 3 | 1.10% | *rps19* | LSC | coding region |
| 825 | 1 | 0.10% | *rpl2* | IRb | coding region |
| 6381 | 39 | 0.60% | *ycf2* | IRb | coding region |
| 2244 | 20 | 0.90% | *ndhF* | SSC | coding region |
| 165 | 1 | 0.60% | *rpl32* | SSC | coding region |
| 978 | 10 | 1% | *ccsA* | SSC | coding region |
| 1530 | 5 | 0.30% | *ndhD* | SSC | coding region |
| 324 | 1 | 0.31% | *ndhE* | SSC | coding region |
| 531 | 2 | 0.40% | *ndhG* | SSC | coding region |
| 1092 | 4 | 0.40% | *ndhA* | SSC | coding region |
| 1182 | 4 | 0.34% | *ndhH* | SSC | coding region |
| 273 | 2 | 0.73% | *rps15* | SSC | coding region |
| 5499 | 129 | 2.34% | *ycf1* | SSC | coding region |
| 392 | 131 | 33.40% | *trnH*(GUG)*-psbA* | LSC | noncoding region |
| 212 | 2 | 0.94% | *psbA-trnK*(UUU) | LSC | noncoding region |
| 1005 | 15 | 1.50% | *trnK*(UUU)*-matk* | LSC | noncoding region |
| 667 | 193 | 28.90% | *trnK*(UUU)*-rps16* | LSC | noncoding region |
| 873 | 9 | 1.00% | *rps16intron* | LSC | noncoding region |
| 1517 | 30 | 2.00% | *rps16-trnQ*(UUG) | LSC | noncoding region |
| 373 | 23 | 6.20% | *trnQ*(UUG)*-psbK* | LSC | noncoding region |
| 378 | 5 | 1.30% | *psbK-psbI* | LSC | noncoding region |
| 139 | 1 | 0.72% | *psbI-trnS*(GCU) | LSC | noncoding region |
| 569 | 15 | 2.60% | *trnS*(GCU)*-trnG*(GCC) | LSC | noncoding region |
| 234 | 34 | 14.50% | *trnG*(GCC)*-trnR*(UCU) | LSC | noncoding region |
| 112 | 3 | 2.70% | *trnR*(UCU)*-atpA* | LSC | noncoding region |
| 717 | 3 | 0.42% | *atpFintron* | LSC | noncoding region |
| 463 | 90 | 19.40% | *atpF-atpH* | LSC | noncoding region |
| 1168 | 26 | 2.20% | *atpH-atpI* | LSC | noncoding region |
| 364 | 95 | 26.10% | *atpI-rps2* | LSC | noncoding region |
| 226 | 7 | 3.10% | *rps2-rpoC2* | LSC | noncoding region |
| 268 | 73 | 27.20% | *rpoC2-rpoC1* | LSC | noncoding region |
| 792 | 49 | 6.20% | *rpoC1intron* | LSC | noncoding region |
| 1262 | 26 | 2.10% | *rpoB-trnC*(GCA) | LSC | noncoding region |
| 694 | 15 | 2.20% | *trnC*(GCA)*-petN* | LSC | noncoding region |
| 1289 | 245 | 19% | *petN-psbM* | LSC | noncoding region |
| 717 | 28 | 3.90% | *psbM-trnD*(GUC) | LSC | noncoding region |
| 120 | 2 | 1.70% | *trnD*(GUC)*-trnY*(GUA) | LSC | noncoding region |
| 796 | 207 | 26% | *trnE*(UUC)*-trnT*(GGU) | LSC | noncoding region |
| 1484 | 114 | 7.70% | *trnT*(GGU)*-psbD* | LSC | noncoding region |
| 225 | 1 | 0.40% | *psbC-trnS*(UGA) | LSC | noncoding region |
| 396 | 38 | 9.60% | *trnS*(UGA)*-psbZ* | LSC | noncoding region |
| 310 | 9 | 2.90% | *psbZ-trnG*(UCC) | LSC | noncoding region |
| 185 | 2 | 1.10% | *trnG*(UCC)*-trnfM*(CAU) | LSC | noncoding region |
| 708 | 5 | 0.71% | *psaA-ycf3* | LSC | noncoding region |
| 1511 | 33 | 2.20% | *ycf3intron* | LSC | noncoding region |
| 808 | 139 | 17.20% | *ycf3-trnS*(GGA) | LSC | noncoding region |
| 323 | 6 | 1.90% | *trnS*(GGA)*-rps4* | LSC | noncoding region |
| 339 | 5 | 1.50% | *rps4-trnT*(UGU) | LSC | noncoding region |
| 878 | 100 | 11.40% | *trnT*(UGU)*-trnL*(UAA) | LSC | noncoding region |
| 367 | 14 | 3.80% | *trnL*(UAA)*-trnF*(GAA) | LSC | noncoding region |
| 379 | 9 | 2.40% | *trnF*(GAA)*-ndhJ* | LSC | noncoding region |
| 112 | 3 | 2.70% | *ndhJ-ndhK* | LSC | noncoding region |
| 1272 | 140 | 11.10% | *ndhC-trnV*(UAC) | LSC | noncoding region |
| 174 | 1 | 0.60% | *trnV*(UAC)*-trnM*(CAU) | LSC | noncoding region |
| 768 | 7 | 0.90% | *atpB-rbcL* | LSC | noncoding region |
| 614 | 6 | 1% | *rbcL-accD* | LSC | noncoding region |
| 442 | 46 | 10.40% | *accD-psaI* | LSC | noncoding region |
| 515 | 67 | 13.10% | *psaI-ycf4* | LSC | noncoding region |
| 874 | 225 | 25.70% | *ycf4-cemA* | LSC | noncoding region |
| 225 | 12 | 5.30% | *cemA-petA* | LSC | noncoding region |
| 1087 | 180 | 16.60% | *petA-psbJ* | LSC | noncoding region |
| 132 | 1 | 0.76% | *psbJ-psbL* | LSC | noncoding region |
| 1019 | 58 | 5.70% | *psbE-petL* | LSC | noncoding region |
| 142 | 12 | 8.50% | *petG-trnW*(CCA) | LSC | noncoding region |
| 152 | 5 | 3.30% | *trnW*(CCA)*-trnP*(UGG) | LSC | noncoding region |
| 406 | 12 | 3% | *trnP*(UGG*)-psaJ* | LSC | noncoding region |
| 422 | 22 | 5.20% | *psaJ-rpl33* | LSC | noncoding region |
| 201 | 16 | 8% | *rpl33-rps18* | LSC | noncoding region |
| 232 | 1 | 0.40% | *rps18-rpl20* | LSC | noncoding region |
| 680 | 9 | 1.30% | *rpl20* | LSC | noncoding region |
| 71676 | 223 | 0.30% | *rps12intron* | LSC | noncoding region |
| 1371 | 19 | 1.40% | *clpPintron* | LSC | noncoding region |
| 453 | 3 | 0.70% | *clpP-psbB* | LSC | noncoding region |
| 194 | 1 | 0.52% | *psbB-psbT* | LSC | noncoding region |
| 85 | 1 | 1.20% | *psbT-psbN* | LSC | noncoding region |
| 128 | 1 | 0.80% | *psbH-petB* | LSC | noncoding region |
| 772 | 28 | 3.60% | *petBintron* | LSC | noncoding region |
| 184 | 1 | 0.50% | *petB-petD* | LSC | noncoding region |
| 764 | 4 | 0.50% | *petDintron* | LSC | noncoding region |
| 117 | 1 | 0.90% | *rps11-rpl36* | LSC | noncoding region |
| 116 | 1 | 0.90% | *rpl36-infA* | LSC | noncoding region |
| 245 | 39 | 15.90% | *rps8-rpl14* | LSC | noncoding region |
| 146 | 9 | 6.20% | *rpl14-rpl16* | LSC | noncoding region |
| 955 | 11 | 1.20% | *rpl16intron* | LSC | noncoding region |
| 166 | 3 | 1.80% | *rpl16-rps3* | LSC | noncoding region |
| 76 | 8 | 10.50% | *rpl22-rps19* | LSC | noncoding region |
| 77 | 20 | 26% | *rps19-rpl2* | IRb | noncoding region |
| 651 | 2 | 0.30% | *rpl2intron* | IRb | noncoding region |
| 669 | 1 | 0.15% | *ycf2-trnL*(CAA) | IRb | noncoding region |
| 688 | 1 | 0.15% | *ndhBintron* | IRb | noncoding region |
| 299 | 1 | 0.33% | *ndhB-rps7* | IRb | noncoding region |
| 2703 | 42 | 1.60% | *rps7-trnV*(GAC) | IRb | noncoding region |
| 1854 | 42 | 2.30% | *rps12-trnV*(GAC) | IRb | noncoding region |
| 585 | 8 | 1.40% | *trnR*(ACG)*-trnN*(GUU) | IRb | noncoding region |
| 2140 | 149 | 7% | *trnN*(GUU)*-ndhF* | IRb | noncoding region |
| 1124 | 185 | 16.50% | *ndhF-rpl32* | SSC | noncoding region |
| 965 | 79 | 8.20% | *rpl32-trnL*(UAG) | SSC | noncoding region |
| 87 | 1 | 1.10% | *trnL*(UAG)*-ccsA* | SSC | noncoding region |
| 224 | 35 | 15.60% | *ccsA-ndhD* | SSC | noncoding region |
| 94 | 7 | 7.40% | *ndhD-psaC* | SSC | noncoding region |
| 266 | 2 | 0.80% | *psaC-ndhE* | SSC | noncoding region |
| 200 | 3 | 1.50% | *ndhE-ndhG* | SSC | noncoding region |
| 440 | 65 | 14.80% | *ndhG-ndhI* | SSC | noncoding region |
| 1091 | 17 | 1.60% | *ndhAintron* | SSC | noncoding region |
| 107 | 17 | 15.90% | *ndhH-rps15* | SSC | noncoding region |
| 404 | 12 | 3% | *rps15-ycf1* | SSC | noncoding region |

**Table S11:** The AT contents of gene in coding and noncoding regions.

| Genes | Regions | AT contents |
| --- | --- | --- |
| *trnH*(GUG) | coding region | 42.70% |
| *psbA* | coding region | 58.00% |
| *trnK*(UUU) | coding region | 45.80% |
| *matk* | coding region | 64.90% |
| *rps16* | coding region | 64.30% |
| *trnQ*(UUG) | coding region | 41.70% |
| *psbk* | coding region | 62.90% |
| *psbI* | coding region | 64.00% |
| *trnS*(GCU) | coding region | 47.70% |
| *trnG*(GCC) | coding region | 48.40% |
| *trnR*(UCU) | coding region | 56.90% |
| *atpA* | coding region | 59.40% |
| *atpF* | coding region | 60.40% |
| *atpH* | coding region | 55.30% |
| *atpI* | coding region | 61.60% |
| *rps2* | coding region | 62.00% |
| *rpoC2* | coding region | 62.50% |
| *rpoC1* | coding region | 61.50% |
| *rpoB* | coding region | 60.70% |
| *trnC*(GCA) | coding region | 42.30% |
| *psbM* | coding region | 71.80% |
| *trnD*(GUC) | coding region | 36.50% |
| *trnY*(GUA) | coding region | 45.20% |
| *trnE*(UUC) | coding region | 43.80% |
| *trnT*(GGU) | coding region | 50.00% |
| *trnS*(UGA) | coding region | 50.50% |
| *trnG*(UCC) | coding region | 47.90% |
| *trnfM*(CAU) | coding region | 43.20% |
| *psbD* | coding region | 57.30% |
| *psbC* | coding region | 55.90% |
| *trnS*(UGA) | coding region | 49.50% |
| *psbZ* | coding region | 65.60% |
| *rps14* | coding region | 57.10% |
| *psaB* | coding region | 58.80% |
| *psaA* | coding region | 57.40% |
| *ycf3* | coding region | 64.20% |
| *trnS*(GGA) | coding region | 48.30% |
| *ndhJ* | coding region | 64.20% |
| *rps4* | coding region | 59.90% |
| *trnT*(UGU) | coding region | 47.40% |
| *trnL*(UAA) | coding region | 52.90% |
| *trnF*(GAA) | coding region | 47.90% |
| *ndhJ* | coding region | 61.20% |
| *ndhK* | coding region | 61.40% |
| *ndhC* | coding region | 65.80% |
| *trnV*(UAC) | coding region | 50.00% |
| *trnM*(CAU) | coding region | 57.50% |
| *atpE* | coding region | 60.00% |
| *atpB* | coding region | 57.20% |
| *rbcL* | coding region | 55.50% |
| *accD* | coding region | 64.80% |
| *psaI* | coding region | 63.10% |
| *ycf4* | coding region | 58.60% |
| *cemA* | coding region | 67.00% |
| *petA* | coding region | 59.30% |
| *psbJ* | coding region | 62.60% |
| *psbI* | coding region | 64.10% |
| *psbF* | coding region | 56.70% |
| *psbE* | coding region | 59.60% |
| *psbL* | coding region | 61.50% |
| *petG* | coding region | 64.20% |
| *trnW*(CCA) | coding region | 48.60% |
| *trnP*(UGG) | coding region | 51.40% |
| *psaJ* | coding region | 60.50% |
| *rpl33* | coding region | 62.90% |
| *rps18* | coding region | 63.10% |
| *rpl20* | coding region | 65.70% |
| *rps12* | coding region | 56.90% |
| *clpP* | coding region | 58.60% |
| *psbB* | coding region | 55.50% |
| *psbT* | coding region | 67.60% |
| *psbN* | coding region | 55.30% |
| *psbH* | coding region | 63.50% |
| *petB* | coding region | 62.60% |
| *petD* | coding region | 63.50% |
| *rpoA* | coding region | 64.10% |
| *rps11* | coding region | 54.20% |
| *rpl36* | coding region | 64.90% |
| *infA* | coding region | 63.70% |
| *rps8* | coding region | 64.70% |
| *rpl14* | coding region | 58.50% |
| *rpl16* | coding region | 65.60% |
| *rps3* | coding region | 65.00% |
| *rpl22* | coding region | 65.30% |
| *rps19* | coding region | 65.20% |
| *rpl2* | coding region | 58.10% |
| *rpl23* | coding region | 62.40% |
| *ycf2* | coding region | 62.00% |
| *trnL*(CAA) | coding region | 51.90% |
| *ndhB* | coding region | 62.50% |
| *rps7* | coding region | 59.60% |
| *trnV*(GAC) | coding region | 51.40% |
| *rrn16* | coding region | 43.50% |
| *trnL*(GAU) | coding region | 40.30% |
| *trnA*(UGC) | coding region | 43.80% |
| *rrn23* | coding region | 45.20% |
| *rrn4.5* | coding region | 49.50% |
| *rrn5* | coding region | 47.90% |
| *trnR*(ACG) | coding region | 37.80% |
| *trnN*(GUU) | coding region | 44.40% |
| *ndhF* | coding region | 68.20% |
| *rpl32* | coding region | 66.10% |
| *trnL*(UAG) | coding region | 42.50% |
| *ccsA* | coding region | 67.20% |
| *ndhD* | coding region | 63.50% |
| *psaC* | coding region | 56.90% |
| *ndhE* | coding region | 68.20% |
| *ndhG* | coding region | 63.50% |
| *ndhI* | coding region | 65.70% |
| *ndhA* | coding region | 64.70% |
| *ndhH* | coding region | 61.60% |
| *rps15* | coding region | 64.80% |
| *ycf1* | coding region | 69.30% |
| *trnH*(GUG)*-psbA* | noncoding region | 75.50% |
| *psbA-trnK*(UUU) | noncoding region | 67.40% |
| *trnK*(UUU)*-matk* | noncoding region | 68.00% |
| *trnK*(UUU)*-rps16* | noncoding region | 70.80% |
| *rps16intron* | noncoding region | 65.70% |
| *rps16-trnQ*(UUG) | noncoding region | 71.00% |
| *trnQ*(UUG)*-psbK* | noncoding region | 72.40% |
| *psbK-psbI* | noncoding region | 69.60% |
| *psbI-trnS*(GCU) | noncoding region | 74.10% |
| *trnS*(GCU)*-trnG*(GCC) | noncoding region | 68.80% |
| *trnG*(GCC)*-trnR*(UCU) | noncoding region | 78.60% |
| *trnR*(UCU)*-atpA* | noncoding region | 74.50% |
| *atpA-atpF* | noncoding region | 72.20% |
| *atpFintron* | noncoding region | 66.80% |
| *atpF-atpH* | noncoding region | 66.10% |
| *atpH-atpI* | noncoding region | 67.80% |
| *atpI-rps2* | noncoding region | 75.70% |
| *rps2-rpoC2* | noncoding region | 69.30% |
| *rpoC2-rpoC1* | noncoding region | 61.60% |
| *rpoC1intron* | noncoding region | 62.80% |
| *rpoB-trnC*(GCA) | noncoding region | 71.60% |
| *trnC*(GCA)*-petN* | noncoding region | 66.40% |
| *petN-psbM* | noncoding region | 71.20% |
| *psbM-trnD*(GUC) | noncoding region | 67.40% |
| *trnD*(GUC)*-trnY*(GUA) | noncoding region | 62.50% |
| *trnY*(GUA)*-trnE*(UUC) | noncoding region | 60.00% |
| *trnE*(UUC)*-trnT*(GGU) | noncoding region | 80.00% |
| *trnT*(GGU)*-psbD* | noncoding region | 69.00% |
| *psbC-trnS*(UGA) | noncoding region | 67.60% |
| *trnS*(UGA)*-psbZ* | noncoding region | 69.00% |
| *psbZ-trnG*(UCC) | noncoding region | 67.20% |
| *trnG*(UCC)*-trnfM*(CAU) | noncoding region | 67.60% |
| *trnfM*(CAU)*-rps14* | noncoding region | 65.20% |
| *psaB-psaA* | noncoding region | 56.00% |
| *rps14-psaB* | noncoding region | 64.20% |
| *psaA-ycf3* | noncoding region | 67.20% |
| *ycf3intron* | noncoding region | 67.50% |
| *ycf3-trnS*(GGA) | noncoding region | 67.30% |
| *trnS*(GGA)*-rps4* | noncoding region | 65.90% |
| *rps4-trnT*(UGU) | noncoding region | 73.70% |
| *trnT*(UGU)*-trnL*(UAA) | noncoding region | 72.80% |
| *trnL*(UAA) *intron* | noncoding region | 64.90% |
| *trnL*(UAA)*-trnF*(GAA) | noncoding region | 66.10% |
| *trnF*(GAA)*-ndhJ* | noncoding region | 73.60% |
| *ndhJ-ndhK* | noncoding region | 68.20% |
| *ndhK-ndhC* | noncoding region | 72.90% |
| *ndhC-trnV*(UAC) | noncoding region | 69.80% |
| *trnV*(UAC)*intron* | noncoding region | 62.10% |
| *trnV*(UAC)*-trnM*(CAU) | noncoding region | 69.00% |
| *trnM*(CAU)*-atpE* | noncoding region | 71.00% |
| *atpB-rbcL* | noncoding region | 70.80% |
| *rbcL-accD* | noncoding region | 71.60% |
| *accD-psaI* | noncoding region | 74.40% |
| *psaI-ycf4* | noncoding region | 65.20% |
| *ycf4-cemA* | noncoding region | 72.90% |
| *cemA-petA* | noncoding region | 71.50% |
| *petA-psbJ* | noncoding region | 69.80% |
| *psbJ-psbL* | noncoding region | 59.10% |
| *psbF-psbE* | noncoding region | 22.20% |
| *psbE-petL* | noncoding region | 71.20% |
| *petL-petG* | noncoding region | 71.20% |
| *petG-trnW*(CCA) | noncoding region | 69.60% |
| *trnW*(CCA)*-trnP*(UGG) | noncoding region | 68.90% |
| *trnP*(UGG)*-psaJ* | noncoding region | 70.60% |
| *psaJ-rpl33* | noncoding region | 68.80% |
| *rpl33-rps18* | noncoding region | 72.40% |
| *rps18-rpl20* | noncoding region | 68.90% |
| *rpl20-rps12* | noncoding region | 67.40% |
| *rps12intron* | noncoding region | 60.50% |
| *clpPintron* | noncoding region | 69.60% |
| *clpP-psbB* | noncoding region | 69.20% |
| *psbB-psbT* | noncoding region | 67.20% |
| *psbT-psbN* | noncoding region | 67.40% |
| *psbN-psbH* | noncoding region | 88.90% |
| *psbH-petB* | noncoding region | 68.50% |
| *petBintron* | noncoding region | 66.00% |
| *petB-petD* | noncoding region | 71.90% |
| *petDintron* | noncoding region | 64.90% |
| *petD-rpoA* | noncoding region | 72.60% |
| *rpoA-rps11* | noncoding region | 82.40% |
| *rps11-rpl36* | noncoding region | 66.70% |
| *rpl36-infA* | noncoding region | 69.80% |
| *infA-rps8* | noncoding region | 59.50% |
| *rps8-rpl14* | noncoding region | 73.90% |
| *rpl14-rpl16* | noncoding region | 69.30% |
| *rpl16intron* | noncoding region | 68.70% |
| *rpl16-rps3* | noncoding region | 72.30% |
| *rpl22-rps19* | noncoding region | 81.90% |
| *rps19-rpl2* | noncoding region | 80.50% |
| *rpl2intron* | noncoding region | 61.30% |
| *rpl2-rpl23* | noncoding region | 77.80% |
| *rpl23-trnL*(CAU) | noncoding region | 66.10% |
| *trnL*(CAU)*-ycf2* | noncoding region | 64.80% |
| *ycf2-trnL*(CAA) | noncoding region | 56.50% |
| *trnL*(CAA)*-ndhB* | noncoding region | 62.90% |
| *ndhBintron* | noncoding region | 61.00% |
| *ndhB-rps7* | noncoding region | 65.10% |
| *rps12-trnV*(GAC) | noncoding region | 59.90% |
| *trnV*(GAC)*-rrn16* | noncoding region | 53.30% |
| *rrn16-trnL*(GAU) | noncoding region | 49.30% |
| *trnL*(GAU)*intron* | noncoding region | 50.90% |
| *trnL*(GAU)*-trnA*(UGC) | noncoding region | 46.90% |
| *trnA*(UGC)*intron* | noncoding region | 49.00% |
| *trnA*(UGC)*-rrn23* | noncoding region | 58.60% |
| *rrn23-rrn4.5* | noncoding region | 41.80% |
| *rrn4.5-rrn5* | noncoding region | 55.90% |
| *rrn5-trnR*(ACG) | noncoding region | 57.40% |
| *trnR*(ACG)*-trnN*(GUU) | noncoding region | 58.00% |
| *trnN*(GUU)*-ndhF* | noncoding region | 64.40% |
| *ndhF-rpl32* | noncoding region | 76.40% |
| *rpl32-trnL*(UAG) | noncoding region | 72.60% |
| *trnL*(UAG)*-ccsA* | noncoding region | 73.60% |
| *ccsA-ndhD* | noncoding region | 70.40% |
| *ndhD-psaC* | noncoding region | 68.20% |
| *psaC-ndhE* | noncoding region | 74.80% |
| *ndhE-ndhG* | noncoding region | 73.50% |
| *ndhG-ndhI* | noncoding region | 75.00% |
| *ndhI-ndhA* | noncoding region | 71.00% |
| *ndhAintron* | noncoding region | 68.50% |
| *ndhH-rps15* | noncoding region | 68.20% |
| *rps15-ycf1* | noncoding region | 74.50% |
